# Uncovering genes driving developmental stage progression in prostate cancer through spatial transcriptomics

**DOI:** 10.1016/j.gendis.2025.101983

**Published:** 2025-12-11

**Authors:** Yongjun Quan, Mingdong Wang, Fan Zou, Hong Zhang, Yishan Zhang, Yongchen Jin, Hao Ping

**Affiliations:** a Department of Urology, Beijing Tongren Hospital, Capital Medical University, Beijing 100176, China; b Department of Pathology, Beijing Tongren Hospital, Capital Medical University, Beijing 100176, China

**Keywords:** Glandular epithelial (GE) cells, Gleason score (GS), H2AFJ, Prostate cancer (PCa), SLC4A4, Spatial transcriptomics (ST)

## Abstract

Precisely delineating the transcriptomic profiles of glandular epithelial (GE) cells in prostate cancer (PCa) remains a significant challenge primarily due to their diffuse and multifocal distribution. To address this, we employed spatial transcriptomics (ST) to analyze 12 PCa tissue samples from 10 patients, aiming to identify PCa progression-associated genes by analyzing expression patterns across histologically distinct regions. Transcriptomic classification via principal component analysis (PCA), uniform manifold approximation and projection (UMAP), and Louvain clustering revealed spatially resolved histological structures within each tissue section. The malignancy status, progression stages, and developmental trajectories of GE clusters were further assessed using inferred copy number variation (inferCNV), diffusion pseudotime (DPT), and partition-based graph abstraction (PAGA) analyses. Based on the preliminary characterization of developmental trajectories, pairwise comparisons of GE clusters identified key oncogenes—including TFF3, OR51E2 (PSGR), FOLH1 (PSMA), AMACR (P504S), FOS (a subunit of AP-1), SLC4A4, EGR1, NDUFB9, and H2AFJ—that are positively associated with PCa progression. Immunohistochemistry (IHC) validation further confirmed the elevated expression of SLC4A4 and H2AFJ in advanced-stage PCa. Overall, this study establishes an ST-based framework for predicting PCa progression and provides valuable insight for the identification of progression-associated genes holding promise as clinical biomarkers.

## Introduction

In 2025, prostate cancer (PCa) is estimated to account for 313,780 new cases and 35,770 deaths in the United States, establishing it as the most prevalent cancer and the second leading cause of cancer-related mortality among men.^1^ These statistics underscore the substantial public health burden posed by PCa and emphasize the urgent need for further research to elucidate its underlying molecular mechanisms and to advance the development of more effective therapeutic strategies.

PCa is widely recognized as a multifocal disease that typically originates within a single gland.^2^^,^^3^ Glandular epithelial (GE) cells, including basal, luminal, and neuroendocrine cells, have been identified as the origin of PCa.^4^, ^5^, ^6^, ^7^, ^8^, ^9^, ^10^, ^11^ Within prostate tissue, PCa frequently exhibits a diffuse distribution of multiple foci, which are often interspersed with non-glandular components, such as the fibromuscular stroma. The intratumor heterogeneity observed in multifocal PCa is hypothesized to arise from either a shared ancestor clone^12^, ^13^, ^14^, ^15^, ^16^ or independent multiclonal origins.^17^, ^18^, ^19^, ^20^ Each lesion may represent a distinct stage in the evolutionary trajectory of PCa. Consequently, analyzing genetic patterns across multiple lesions is essential for elucidating the origins of PCa and identifying key drivers of its progression.

Accurately delineating the gene expression profiles of GE cells in PCa remains challenging due to the poorly defined macroscopic boundaries between tumor and adjacent non-tumor tissues. Although single-cell sequencing (scRNA-seq) enables transcriptomic profiling at the cellular level,^21^, ^22^, ^23^ this approach is limited by its reliance on computational inference and the lack of spatial context, which is essential for resolving the complex histological architecture of PCa and its surrounding microenvironment.^24^^,^^25^

Spatial transcriptomics (ST) overcomes these limitations by integrating RNA sequencing with spatial localization, thereby allowing direct correlation of transcriptomic profiles with specific histological features and tissue morphology.^16^^,^^26^, ^27^, ^28^, ^29^, ^30^, ^31^, ^32^ The Visium ST platform achieves high spatial resolution through microarrays with 4992 spatially barcoded spots (each 55 μm in diameter) distributed over a 6.5 × 6.5 mm^2^ capture area. The Visium CytAssist ST is an advanced technology that further enhances accuracy in gene expression mapping by employing precise tissue transfer and multiple specific probe pairs to capture high-quality target sequences with reduced background noise.^27^^,^^30^ This improvement enables robust integration of spatial transcriptomic data with distinct pathological structures, thereby providing deeper insights into spatial gene activity and underlying biological processes.^16^^,^^29^^,^^31^, ^32^, ^33^, ^34^, ^35^, ^36^

Prior to analyzing multifocal PCa, it is essential to conduct a standardized evaluation of the progression stage for each lesion based on hematoxylin-eosin (HE)-stained images. The Gleason grading system remains the most reliable approach for assessing the pathological stage and progression of PCa.^37^, ^38^, ^39^, ^40^ This system enables the prediction of PCa malignancy and offers critical prognostic insights, including the risk of extraprostatic extension, biochemical recurrence, and overall survival.^41^, ^42^, ^43^, ^44^, ^45^, ^46^ The Gleason score (GS), which is derived from the primary and secondary Gleason patterns, ranges from 1 + 1 to 5 + 5. PCa patients with a GS sum of 7 or higher are classified as having aggressive disease, which is associated with a high risk of extraprostatic extension and an unfavorable prognosis.^47^^,^^48^

In our previous research, we performed preliminary Visium ST analysis on a limited number of PCa tissue samples to identify genes potentially associated with PCa progression.^29^^,^^31^^,^^32^ These candidate genes were subsequently validated through bioinformatic databases, histopathological assessments, and cellular functional experiments.^29^^,^^31^^,^^32^ However, the restricted sample size in these earlier investigations may have limited the ability to fully capture inter-individual heterogeneity, potentially introducing inaccuracies into the findings. In the present study, we expanded the scope of analysis to include 12 PCa tissue samples obtained from 10 patients, employing both Visium and Visium CytAssist ST platforms to investigate transcriptome expression patterns across PCa tissues exhibiting diverse GE clusters. Key genes linked to GS progression were identified and further validated through immunohistochemistry (IHC) in an independent cohort of 90 PCa tissue samples.

## Materials and methods

### Collection of prostate cancer (PCa) tissues

A cohort of ninety PCa patients who had undergone prostatectomy was recruited for this study. Their clinicopathological data are detailed in Table 1. Both PCa and prostatic intraepithelial neoplasia (PIN) tissues from these patients were extracted and stored into the 10% neutral formalin, and then embedded in paraffin for subsequent IHC analysis.

**Table 1:** Clinicopathological characteristics of prostate cancer patients for IHC analysis.

Clinicopathological parameters	Total (*n* = 90)
**Age**	
Median age, year (IQR)	68.5 (65–73.25)
Range, year (Min, Max)	54–83
**Number of age category, *n* (%)**	
<65	19 (21.1)
≥65	71 (78.9)
**Pre-RP t-PSA level**	
Median, ng/mL (IQR)	9.65 (5.66–21.375)
Range, ng/mL (Min, Max)	0.1–160
**Number of t-PSA status, *n* (%)**	
<4 ng/mL	8 (8.9)
4–10 ng/mL	38 (42.2)
10–20 ng/mL	20 (22.2)
>20 ng/mL	24 (26.7)
**f-PSA/t-PSA (f/t)**	
Median (IQR)	0.1185 (0.1–0.17)
Range (Min, Max)	0.0–0.5
**Number of f/t status, *n* (%)**	
<0.16	63 (70.0)
≥0.16	27 (30.0)
**Number of total GS status, *n* (%)**	
<7	20 (22.2)
=7	36 (40.0)
>7	34 (37.8)
**Number of pT-stage, *n* (%)**	
T2	53 (58.9)
T3a	18 (20.0)
T3b	11 (12.2)
T4	8 (8.9)
**Number of ISUP grade, *n* (%)**	
1	20 (22.2)
2	20 (22.2)
3	16 (17.8)
4	19 (21.1)
5	15 (16.7)
**Number of AJCC stage, *n* (%)**	
Ⅰ	9 (10.0)
ⅡA	7 (7.8)
ⅡB	16 (17.8)
ⅡC	8 (8.9)
ⅢA	11 (12.2)
ⅢB	24 (26.7)
ⅢC	15 (16.7)

IQR: Interquartile range; RP, radical prostatectomy; t-PSA: total prostate-specific antigen; f-PSA: free prostate-specific antigen; GS: Gleason score; pT: pathological T; ISUP: International Association of Urology Pathology; AJCC: American Joint Committee on Cancer.

Eight PCa samples extracted from six patients were subjected to Visium CytAssist spatial transcriptomics (ST) analysis (Table 2). Additionally, Visium ST data from four PCa samples in our previous study^29^ were reanalyzed and integrated into the current dataset. These tissue extraction sites were precisely identified using prostate magnetic resonance imaging (MRI) and confirmed through pathological diagnosis of prostate needle core biopsies. In two cases, bilateral tumors with differing GSs were observed, raising the possibility of independent tumor formation in each lobe; consequently, two samples were collected from each of these tissues. Fresh tissue specimens were snap-frozen on dry ice, embedded in optimal cutting temperature (OCT) compound, and stored at −80 °C until further analysis.

**Table 2:** Clinicopathological characteristics of selected PCa patients for ST analysis.

No.	Age	TPSA (ng/mL)	FPSA/TPSA	GS (pathology)	pT	ISUP grade	AJCC stage
Cytassist_1	68	16.8	0.089	3 + 4	T2	2	ⅡB
Cytassist_2	66	4.5	0.111	4 + 3	T2	3	ⅡC
Cytassist_3	65	27.1	0.192	4 + 3	T3b	3	ⅢB
Cytassist_4							
Cytassist_5	65	40.9	0.071	4 + 3	T4	3	ⅢB
Cytassist_6	69	12.2	0.066	4 + 5	T3a	5	ⅢC
Cytassist_7							
Cytassist_8	66	10.6	0.1	3 + 4	T2	2	ⅡB
Visium_1	65	23.91	0.1	3 + 4	T3b	2	ⅢB
Visium_2	71	32.4	0.259	4 + 4	T3b	4	ⅢB
Visium_3	63	58.88	0.086	5 + 4	T2	5	ⅢC
Visium_4	72	9.86	0.175	4 + 3	T2	3	ⅡC

TPSA: total prostate specific antigen; FPSA: free prostate specific antigen; GS: Gleason score; ISUP: International Association of Urology Pathology; AJCC: American Joint Committee on Cancer.

All the human prostate tissue samples were obtained from Beijing Tongren Hospital. Informed consent was obtained from all patients, and the study was approved by the Ethics Committee of Beijing Tongren Hospital, affiliated with Capital Medical University (Approval No. TREC2023-KY030).

### Visium CytAssist spatial transcriptomics (ST) (10x genomics visium)

**Figure 1:**
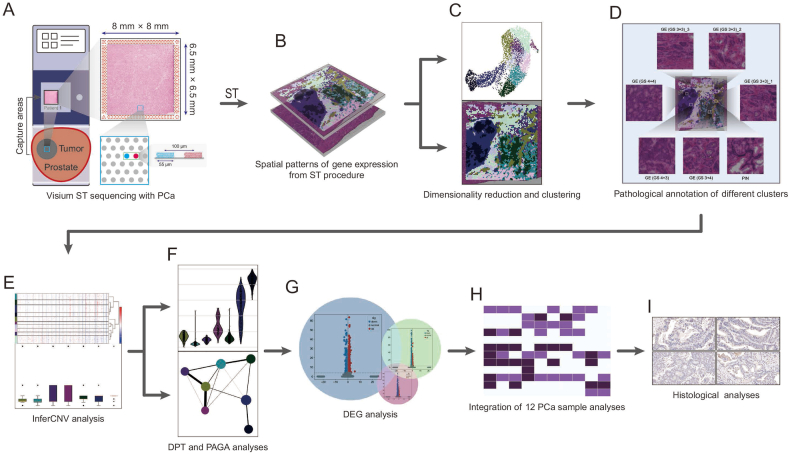
Workflow of this study. **(A)** Cryosectioned prostate cancer (PCa) tissue samples were mounted on Superfrost slides (VWR) containing a spatial microarray of 4992 barcoded spots (55 μm in diameter, 100 μm center-to-center spacing) to enable spatially resolved gene expression profiling. **(B)** Transcriptomic read count matrices were generated following the CytAssist ST platform. **(C)** Dimensionality reduction and clustering of the spatial spots were performed through PCA, UMAP and Louvain clustering analyses. **(D)** The clusters were annotated as distinct histological structures based on pathological evaluation. **(E)** Malignancy-associated features of the classified clusters were assessed through inferCNV analysis. **(F)** Developmental trajectories and progression stages were further characterized using DPT and PAGA analyses. **(G)** To identify genes associated with PCa progression, DEG analysis was conducted by comparing GE clusters aligned with GS progression patterns that met all predefined criteria. **(H)** Commonly dysregulated DEGs across 12 PCa samples were identified and summarized. **(I)** The protein expression of candidate DEGs (H2AFJ, SLC4A4, TFF3) was validated in PCa tissues spanning a spectrum of GSs and PIN using IHC staining.

Tissue sections embedded in OCT with an RNA integrity number (RIN) ≥ 4 were cryosectioned at a thickness of 10 μm and mounted on Superfrost slides (VWR, Radnor, Pennsylvania, USA) according to Demonstrated Protocol CG000636. The spatial microarray on the slide contained 4992 distinct barcoded spots within capture areas measuring 6.5 × 6.5 mm^2^. Each spot had a diameter of 55 μm and a center-to-center distance of 100 μm, providing transcriptome information representing the gene expression within the tissue (Fig. 1A). Tissue sections mounted on the slides were fixed in pre-cooled methanol solution at −20 °C for 30 min, followed by HE staining and microphotography using the Olympus BX53 microscope (Olympus, Shinjuku-ku, Tokyo, Japan) and were subsequently destained as per Demonstrated Protocol CG000614. Probe hybridization, wash, ligation, and release procedures were then performed on the destained tissue slides. Next, the slides with tissue sections were processed using the Visium CytAssist instrument (10x Genomics, CA, USA), during which the probes were transferred to the capture area of the Visium CytAssist spatial gene expression slide. Probe extension, pre-amplification, and library construction were completed following the Visium CytAssist Spatial Gene Expression Reagent Kits User Guide (CG000495). Sequencing was conducted on the Illumina NovaSeq X Plus platform, with sequencing libraries demultiplexed with bcl2fastq (Illumina, CA, USA). The resulting FASTQ files were aligned to the human reference genome (GRCh38) using Space Ranger v3.0.

### Analysis of gene expression using scanpy package in python

The transcriptomic profiling data generated by Space Ranger were analyzed using the Python (version 3.12) package Scanpy. In the data filtering phase, a maximum threshold for mitochondrial gene proportion was assigned to each sample based on its highest observed value (rounded to two decimal places), a strategy designed to incorporate virtually all spatial spots with detectable gene expression. The read count data were normalized to ensure consistent total expression across all spatial spots and subsequently log-transformed using the natural logarithm [ln (1 + normalized value)] to improve the distribution normality and mitigate the impact of highly expressed genes. Highly variable genes (HVGs) were identified using the following thresholds: a mean expression range of 0.0125–3 and a dispersion range of 0.5–1000. Additionally, an image processing algorithm was applied to the captured region of the PCa tissue section based on spatial barcode information from the pre-processed data and the sequencing reads corresponding to each spatial spot.

### Dimensionality reduction

To reduce the dimensionality of the transcriptomic data from PCa sections, we performed principal component analysis (PCA) using HVGs as input and retained the top 50 principal components for subsequent analysis. The detailed methodology is detailed in the Scikit-learn documentation (https://scikit-learn.org/stable/modules/generated/sklearn.decomposition.PCA.html). Leveraging the PCA-reduced data, we then applied uniform manifold approximation and projection (UMAP) to visualize the global transcriptomic structure in two-dimensional space. Following this, Louvain clustering analysis was employed to categorize the data points into classification ns, allowing for the simultaneous alignment of transcriptomic patterns with histological structures.

### Inferred copy number variation (inferCNV) analysis

The acquisition of specific genomic aberrations has been linked to the progression of PCa to advanced stages.^12^, ^13^, ^14^, ^15^, ^16^^,^^49^^,^^50^ CNV analysis is instrumental in elucidating clonal hierarchies and assessing tumor malignancy.^12^, ^13^, ^14^, ^15^, ^16^^,^^22^^,^^23^ In this study, somatic large-scale chromosomal CNVs were inferred by employing transcriptomic data obtained from ST analysis, facilitated through a Python reimplementation of inferCNV (https://infercnvpy.readthedocs.io/en/latest/infercnv.html). The expression intensity of genes across positions in the whole genome was examined in relation to a designated set of “normal” reference spots. We utilized PIN as the primary reference group. In instances where PIN cells were unavailable, cells with a GS of 3 + 3 were used as an alternative reference group due to their well-differentiated characteristics and low metastatic potential,^51^, ^52^, ^53^ which have been proposed to represent precancerous lesions.^54^^,^^55^ In cases where neither PIN nor GS (3 + 3) were available, the group with the lowest GS was selected as the reference group. The inferCNV values of local genomic fragments across 22 autosomes were visualized in a heatmap, while the median CNV signals within each cluster were depicted in boxplots. This approach was designed to elucidate the cellular origins^12^, ^13^, ^14^, ^15^, ^16^ and malignancy-associated features of each cluster.^22^^,^^23^

### Diffusion pseudotime (DPT) analysis

DPT enables the inference of cellular progression in biological processes from snapshot data by leveraging geodesic distance along the graph structures.^56^ This approach allows for the reconstruction of the developmental progression of PCa cells, facilitating the identification of transient or metastable states, branching decisions and differentiation endpoints.^56^ It is implemented within the Scanpy framework (https://scanpy.readthedocs.io/en/stable/generated/scanpy.tl.dpt.html).^57^

### Partition-based graph abstraction (PAGA) analysis

PAGA is a graph abstraction that reconciles clustering with trajectory inference through a topology preserving map of single cells.^58^ PAGA generates an interpretable, graph-like map (PAGA graph) of the arising data manifold, based on estimating connectivity of manifold partitions (https://github.com/theislab/paga). PAGA maps preserve the global topology of data, enable multi-resolution analysis, and significantly enhance the computational efficiency of the typical exploratory data analysis workflow. It not only captures both discrete disconnected and continuous connected cell-to-cell variations but also reliably predicts developmental trajectories and gene expression dynamics using transcriptomic datasets. The algorithms used to generate PAGA were previously described in a prior study^58^ and have been implemented within the Scanpy framework (https://scanpy.readthedocs.io/en/stable/generated/scanpy.tl.paga.html).

### Differentially expressed gene (DEG) analysis

DEG analysis was conducted to thoroughly explore the heterogeneity of gene expression across various developmental stages of histological clusters in PCa. DEGs between the specified clusters were identified using the Wilcoxon test implemented in scanpy.tl.rank_genes_groups (https://scanpy.readthedocs.io/en/stable/generated/scanpy.tl.rank_genes_groups.html). For this analysis, filtering thresholds were set such that the absolute value of the natural logarithm of the fold change (|ln FC|) > 0.25, and the false discovery rate (FDR) as determined by the Benjamini–Hochberg procedure (Q-value) < 0.0001. The distribution of DEGs across various clusters was summarized and visualized using volcano plots and Venn diagrams.

### Score a set of genes

The composite score for a specific gene set was calculated using the scanpy.tl.score_genes function, as outlined in the Scanpy documentation (https://scanpy.readthedocs.io/en/stable/generated/scanpy.tl.score_genes.html). The score is computed as the difference between the mean expression of a target gene set and that of a reference gene set. The reference set is generated by random sampling from the gene_pool based on binned expression values. This methodology, initially developed in Seurat,^24^ was implemented in Scanpy by Davide Cittaro. The gene score was utilized to comprehensively evaluate the expression patterns of the selected gene sets across various stages of histological structure.

### Enrichment analysis

The biological functions, signaling pathways, and diseases associated with the identified DEGs were elucidated through Gene Ontology (GO) and Kyoto Encyclopedia of Genes and Genomes (KEGG) enrichment analyses. These analyses were conducted using R software (version 4.3.1) with the “clusterProfiler”, “enrichplot”, “org.Hs.eg.db”, and “ggplot2” packages.

### Bioinformatics analysis of the cancer genome Atlas prostate adenocarcinoma (TCGA-PRAD) dataset

The input gene set was validated using data from the TCGA-PRAD cohort. Clinicopathological characteristics and follow-up information were obtained from two primary sources: the Genomic Data Commons (GDC) Data Portal (https://portal.gdc.cancer.gov/) and the UCSC Xena platform (https://xenabrowser.net/datapages/).^59^ These comprehensive datasets provided robust phenotypic and clinical annotations essential for subsequent analyses. Gene expression read counts from TCGA-PRAD patients were normalized to transcripts per kilobase million (TPM) to account for variations in sequencing depth and gene length. Subsequently, key clinicopathological variables, including GS, pathological T stage (pT), and TP53 mutation status, were statistically analyzed using R software (version 4.3.1) with the “limma”, “reshape2”, and “ggpubr” packages. Kaplan–Meier survival analysis and Cox proportional hazards regression were performed to evaluate two prognostic outcomes: recurrence-free survival (RFS) and progression-free survival (PFS). The corresponding RFS and PFS data were extracted from the phenotype files, as previously detailed.^59^^,^^60^ Survival analyses were conducted using R software (version 4.3.1) with the “survival” and “survminer” packages.

### Immunohistochemistry (IHC) analysis

IHC analysis was performed to confirm the protein expression of the representative oncogenes. Paraffin-embedded tissue samples were sectioned into 5 μm slices and subjected to antigen retrieval. The sections were subsequently incubated with specific primary antibodies, followed by the appropriate secondary antibodies. Detailed information on the antibodies used is provided in Table S1.

### Statistical analysis

Statistical analyses were conducted using Python (version 3.12), R software (version 4.3.1), SPSS (version 23; IBM, Armonk, NY, USA), GraphPad Prism (version 9.0.0; GraphPad Software, La Jolla, CA, USA), and Microsoft Excel 2019 (Microsoft Corp., Redmond, WA, USA). Continuous variables were compared between two groups using the Wilcoxon test or Student's *t*-test, along with *P*- or *Q*-value calculations. For comparisons among three or more groups, the Kruskal–Wallis test or one-way/two-way analysis of variance (ANOVA) was applied, followed by *post hoc* tests, including Tukey's test or Dunn's multiple comparison test. Survival analysis was conducted using Kaplan–Meier curves with the log-rank test and univariate Cox regression models to calculate hazard ratios (HRs). *P*-values or *Q*-values less than 0.05 were considered statistically significant.

To quantify the strength of the IHC staining and perform the statistical analysis, representative images were selected at 36.4× magnification. The staining intensity was scored on a scale of 1–3 (1: weak, 2: moderate, 3: strong), and the percentage of positively stained cells was scored as follows: 0 for ≤ 5%, 1 for 6%–25%, 2 for 26%–50%, 3 for 51%–75%, and 4 for ≥ 76%. The staining index (SI) was then calculated as the product of the staining intensity score and the staining percentage score, following previously established methods.^61^^,^^62^

## Results

### Workflow of the Visium CytAssist ST analysis

Figure 1 illustrates the workflow of the spatial transcriptomics analysis in this study. The extracted PCa tissues were cryosectioned and mounted on Superfrost slides (VWR) (Fig. 1A). Transcriptomic profiling matrices were obtained following the Visium CytAssist Spatial Transcriptomics protocol (Fig. 1B). Subsequent dimensionality reduction and Louvain clustering analysis grouped the spots into distinct clusters (Fig. 1C). These clusters were annotated according to histological structures, encompassing GE with varying GSs and PINs, as well as non-glandular tissues (Fig. 1D). InferCNV, DPT, and PAGA were then performed to evaluate malignancy, progression stages, and developmental trajectories across distinct tissue types (Fig. 1E and F). To identify genes associated with PCa progression, DEG analysis was conducted based on comparisons aligned with GS progression patterns (Fig. 1G). Key genes implicated in PCa development were identified and validated using the TCGA-PRAD dataset (Fig. 1H). Functional interpretation was carried out through GO and KEGG enrichment analyses of these candidate genes. Finally, the differential protein expression of selected genes was confirmed via IHC analysis across representative PCa tissues (Fig. 1I).

### The transcriptomic classification of all spatially resolved spots across 12 PCa tissue sections reveals the molecular and spatial heterogeneity of the tissue microenvironment

**Figure 2:**
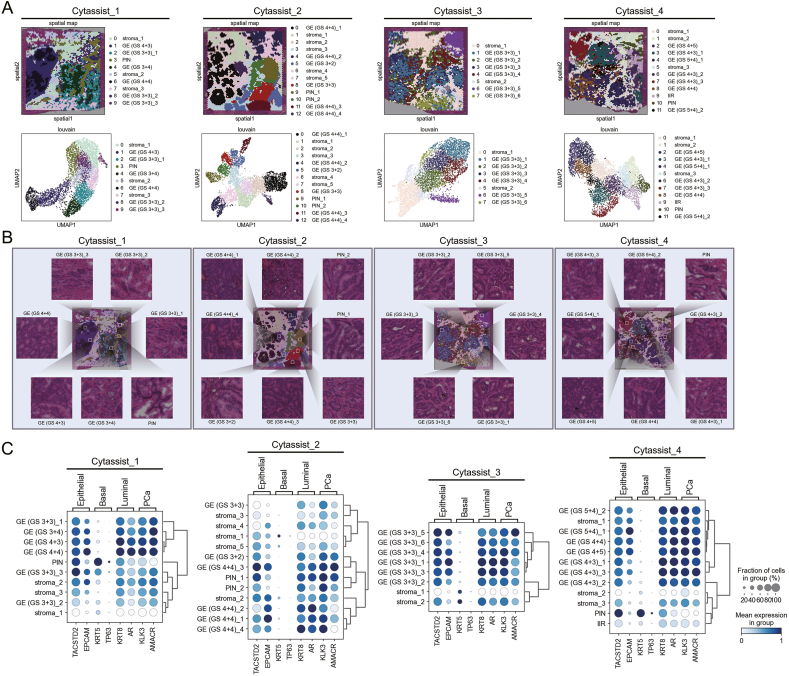
The transcriptomic classification of all spatially resolved spots across CytAssist_1 to CytAssist_4 prostate cancer (PCa) tissue sections into distinct histological structures. **(A)** Transcriptomic classifications across all spots were performed using PCA, UMAP, and Louvain clustering analysis. The resulting clusters were overlaid onto the histological image and annotated according to their corresponding histological structures (upper panel), and visualized in a UMAP embedding (lower panel). **(B)** The regions of distinct clusters were magnified and pathologically evaluated based on corresponding HE-stained images from each PCa section. **(C)** The expression of canonical marker genes—including TACSTD2 and EPCAM (epithelial), KRT5 and TP63 (basal), KRT8 and AR (luminal), and KLK3 and AMACR (PCa)—was analyzed across annotated clusters and visualized as bubble plots.

The transcriptomic expression data from 12 PCa sections were dimensionality reduced using PCA and clustered into distinct groups via Louvain clustering analysis (Fig. 2A; Figs. S1A and S2A). Based on the histological structures observed in HE-stained images, each cluster was pathologically diagnosed and annotated as GSs, PIN, or non-glandular tissues—including fibromuscular stroma, blood vessels (BVs), and immune infiltration regions (IIRs) (Fig. 2B; Figs. S1B and S2B).

Annotation accuracy was validated by assessing the expression of canonical marker genes specific to each cell type: epithelial cells (TACSTD2, EPCAM), basal cells (KRT5, TP63), luminal cells (KRT8, AR), and PCa cells (KLK3, AMACR, and PCA3)^33^ (Fig. 2C; Figs. S1C and S2C). Notably, PCA3 was not detectable in the Visium Cytassist ST data due to limited gene probe coverage and was therefore validated only in the four Visium ST data (Fig. S2C). In the majority of PCa regions across varying GSs, epithelial, luminal, and PCa-specific markers were relatively highly expressed, whereas basal cell markers showed comparatively low expression (Fig. 2C; Figs. S1C and S2C). Conversely, PIN tissues from Cytassis_1, Cytassist_4, and Visium_3 exhibited elevated expression of basal cell markers and reduced levels of PCa-specific markers (Fig. 2C; Fig. S2C), confirming the general concordance between the histological morphology and transcriptomic profiles.

However, certain regions—such as the PIN tissue in Cytassist_2—deviated from expected expression patterns, potentially due to variance-based normalization artifacts or transcriptional contamination from PCa cells mixed within PIN. These discrepancies indicate that marker expression alone may not fully capture histological identity, underscoring the limitations of prior scRNA-seq studies that overrelied on computational annotation without histological correlation.^21^, ^22^, ^23^, ^24^, ^25^

### The malignancy of the GE regions assessed by inferCNV analysis consistent with the GS characteristics

**Figure 3:**
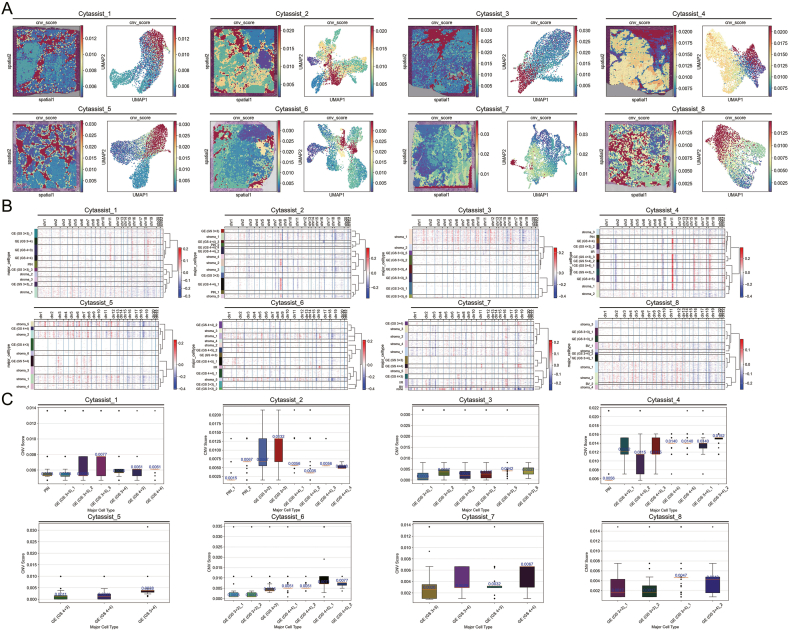
Evaluation of tumor malignancy in varying GE regions across CytAssist_1 to CytAssist_8 prostate cancer (PCa) tissue sections using inferCNV analysis. **(A)** The CNV scores were calculated for spatially resolved spots across eight samples using inferCNV. The scores are visualized as spatial activity maps overlaid on the histological images (left panel) and as UMAP embeddings (right panel). **(B)** The chromosomal landscapes derived from inferCNV values for distinguishing histological clusters across the samples are displayed separately as corresponding heatmaps. **(C)** The median CNV scores, along with their interquartile ranges (IQRs), for GE clusters are summarized using boxplots for each sample.

InferCNV analysis has been demonstrated to be an effective tool for predicting clonal hierarchies and assessing tumor malignancy.^12^, ^13^, ^14^, ^15^, ^16^^,^^22^^,^^23^ The inferCNV values are visualized in the histological images, UMAP embeddings, and chromosomal landscape (Fig. 3A and B; Fig. S3A and S3B). The mean CNV value was used as the primary criterion for assessing the malignancy of the histological clusters.^22^^,^^23^ In this study, we computed both the median and mean inferCNV scores for each histological cluster and selected the most appropriate measure based on the distribution characteristics of the data, including normality, skewness, and the presence of outliers. Assessments of tumor malignancy based on the inferCNV index were consistent with histopathological evaluation and showed a general increase with higher GSs across most GE clusters, although not universally (Fig. 3C; Fig. S3C).

### Prediction of progression stages and developmental trajectories of GE clusters

**Figure 4:**
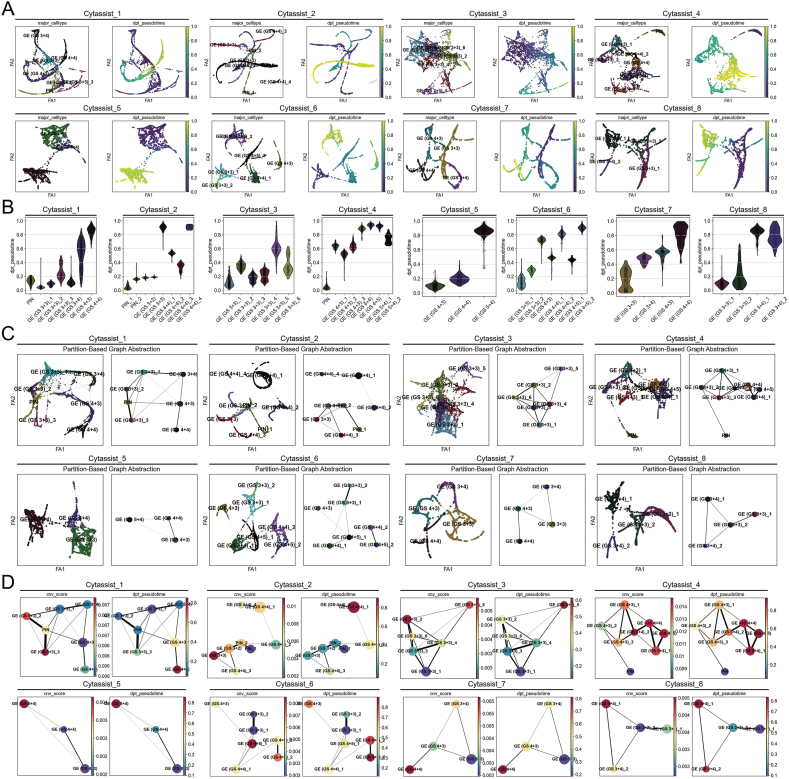
Prediction of progression stages and developmental trajectories across CytAssist_1 to CytAssist_8 prostate cancer (PCa) tissue sections. **(A)** Developmental progression was reconstructed using DPT analysis. Diffusion maps of spatially resolved spots for each sample are presented, displaying coordinates specific to varying GE clusters (left panel) and corresponding dpt_pseudotime values (right panel). **(B)** The quantitative distribution of dpt_pseudotime values within GE clusters for each sample is visualized as violin plots. **(C)** Developmental trajectories of GE clusters for each sample are visualized using PAGA graphs, displaying individual spatial spots (left panel) and GE clusters (right panel). **(D)** The CNV score (left panel) and dpt_pseudotime values (right panel) were integrated into the PAGA graph to illustrate developmental dynamics within each GE cluster.

We employed DPT and PAGA analyses to infer developmental trajectories and progression stages across distinct clusters.^56^^,^^58^ DPT reconstructs cellular developmental progression, identifies branching decisions and differentiation endpoints, and thereby reveals dynamic bifurcation events along differentiation pathways.^56^ Geodesic distances from diffusion maps, combined with dpt_pseudotime values, effectively illustrate both the relational structures and progression ordering among these clusters (Fig. 4A; Fig. S4A). This trajectory collectively delineates a continuous developmental path and highlights transitional states and lineage relationships between GE clusters. Quantitative assessment indicated a general trend of increasing dpt_pseudotime values with advancing GS, although not universally (Fig. 4B; Fig. S4B).

Complementarily, PAGA captures both discrete disconnected and continuous connected cell-to-cell variations, while also reliably predicting developmental trajectories and gene expression changes from transcriptomic datasets.^58^ The static PAGA graphs, which retain each spot and GE cluster, illustrated the associations and potential developmental trajectories among the GS subtypes and PIN (Fig. 4C; Fig. S4C). To comprehensively evaluate the inter-cluster relationships, degree of malignancy, and progression patterns, we integrated the cnv_score and dpt_pseudotime values into the PAGA graph (Fig. 4D; Fig. S4D). This approach enabled us to identify GE clusters that simultaneously satisfied all three predefined criteria in alignment with GS, thereby establishing a foundation for subsequent DEG analysis according to developmental stage.

### DEG analysis based on the developmental progression across varying clusters

DEG analysis was conducted on GE clusters that satisfied all predefined criteria derived from the GS, inferCNV, DPT, and PAGA analyses. Eligible comparisons were required to exhibit consistent relative malignancy levels—as defined by both GS and cnv_score—along with coherent progression stages and developmental trajectories within the dpt_pseudotime values and PAGA graphs.

**Figure 5:**
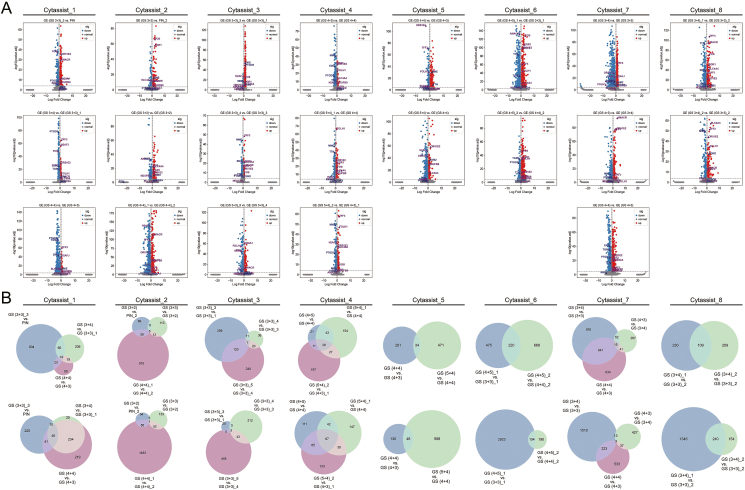
DEG analysis across varying clusters based on progression stages and developmental trajectories in CytAssist_1 to CytAssist_8 prostate cancer (PCa) tissue sections. **(A)** DEG analysis was performed for GE clusters, ensuring that the comparison objects exhibited consistent relative progression stages and developmental trajectories. The comparisons for each tissue section are illustrated using volcano plots, with key genes highlighted: oncogenes (TFF3, OR51E2, FOLH1, AMACR, FOS, SLC4A4, EGR1, NDUFB9, and H2AFJ) in dark magenta and antioncogenes (MME, PTGDS, TTN, ENG, and TGM2) in dark blue. **(B)** The numbers of genes that were significantly and concurrently up-regulated (upper panel) or down-regulated (lower panel) in the above comparisons for each tissue section are depicted in Venn diagrams.

For example, in Cytassis_1, the PAGA graph revealed strong correlations among GS (3 + 3)_3 and PIN, GS (3 + 4) and GS (3 + 3)_1, and GS (4 + 4) and GS (4 + 3) (Fig. 4C and D). These groups also showed concordant trends in GS, cnv_score, and dpt_pseudotime values (Figure 3, Figure 4B). Based on these consistencies, we included these comparisons in the DEG analysis. Significantly differentially expressed genes were defined as those with |ln FC| > 0.25 and *Q*-value < 0.0001 (Wilcoxon test) (Fig. 5A). We then complied genes linked to GS progression that were simultaneously positively (such as NPY, AR, FOXA1, FOLH1, and H2AFJ) or negatively (such as JUNB, CD74, FOS, and PTGDS) regulated across two or three comparisons (Fig. 5B). The same analytical procedure was applied to other samples, with all DEG analyses involving two or three comparative groups (Figure 3, Figure 4, Figure 5; Fig. S3–S5).

In samples such as Cytassis_3 and Visium_4, which exhibited histologically similar GS structures among classified lesions (Fig. 2; Fig. S2), DEG analysis was guided primarily by developmental progression predictions from inferCNV, DPT, and PAGA analyses (Fig. 5B; Fig. S5B).

### Identification of key genes associated with PCa progression across developmental stages

**Figure 6:**
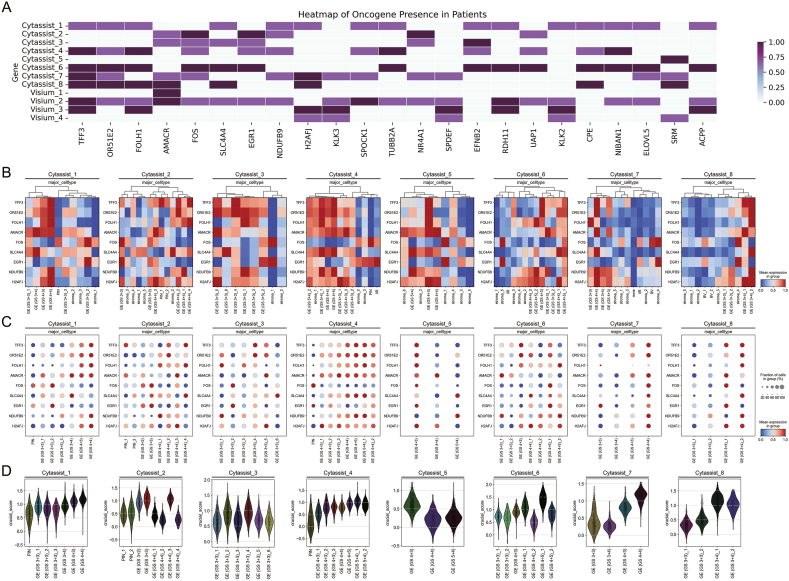
Identifying key oncogenes associated with the progression of developmental stages in 12 prostate cancer (PCa) tissue sections. **(A)** Significant DEGs intersected at least two comparisons within each tissue are summarized and visualized as heatmap. Each gene is assigned a value of 1 (dark purple) if consistently up-regulated across all comparisons within a sample, 0.66 (light purple) if up-regulated in two of three comparisons, and 0 (white) otherwise. **(B)** The expression patterns of oncogenes shared by at least five tissue sections within clusters corresponding to the distinct histological structures of each tissue are visualized as heatmaps. **(C)** The expression patterns of these genes within distinct GE structures of each tissue are displayed as bubble plots. **(D)** Gene scores (crucial_scores) derived from the 9 identified oncogenes were calculated, and their distributions across varying GE clusters in each tissue section are visualized as violin plots.

Significant DEGs that appeared in at least two comparisons within each tissue section were identified. These DEGs exhibited consistent patterns of either high or low expression across developmental stages in PCa tissue sections (Fig. 6A; Fig. S7A). The results indicate that among the genes analyzed, TFF3 was consistently overexpressed in advanced stages across the seven PCa tissues (Fig. 6A). Additionally, several well-established PCa-associated genes, including OR51E2 (prostate-specific G protein-coupled receptor, PSGR), FOLH1 (prostate specific membrane antigen, PSMA), AMACR (P504S), FOS [a subunit of activating protein-1 (AP-1)], KLK3 (prostate-specific antigen, PSA), and KLK2, were found to be overexpressed in the advanced stages of at least four PCa tissues (Fig. 6A).

We then identified genes that were consistently overexpressed (9 oncogenes) or downregulated (5 antioncogenes) in advanced stages across at least five PCa samples. The expression patterns of these genes were further analyzed within the clusters corresponding to the distinct histological structures of each tissue. In most PCa tissues, these genes exhibited a gradual increase or decrease in expression with GS progression (Fig. 6B and C; Fig. S6A and S6B, Fig. S7B and S7C, Fig. S8A and S8B). Gene sores derived from the identified 9 oncogenes and 5 antioncogenes were calculated and subsequently evaluated across varying GSs within each tissue section. The oncogene scores in most tissues generally increased with GS progression, although not universally (such as Cytassist_5), whereas the scores from antioncogenes showed an opposite trend (Fig. 6D; Figs. S6C, S7D, S8C).

Next, we analyzed bilateral lesion samples extracted from the same prostate tissue (Cytassist_3/4 and Cytassist_6/7. Despite originating from the same prostate, each sample pair shared few oncogenes and antioncogenes, revealing significant transcriptomic heterogeneity in multifocal PCa (Fig. S9A and S9B).

### Validation of the identified key genes using the TCGA-PRAD dataset

We subsequently evaluated the genes shared by at least three samples using the TCGA-PRAD dataset. The expression patterns of these genes were analyzed across multiple comparisons based on clinicopathological characteristics, including tumor versus normal tissue, GS > 7 versus GS < 7, pT3 versus pT2, and TP53-mutated versus wild-type tissues. Additionally, survival analyses were conducted to assess the prognostic significance of the gene expression status for RFS and PFS, as previously reported in our studies.^29^^,^^31^^,^^32^^,^^60^^,^^63^ Significant DEGs were defined as having a *P* < 0.05 in either the Wilcoxon test or Cox regression analysis.

The results revealed that several oncogenes (such as FOLH1 and NDUFB9) and antioncogenes (such as MME and PTGDS) exhibited generally consistent expression patterns with the ST analysis at advanced stages (Fig. S10A–S10D, S11A–S11D). However, several well-known oncogenes, including OR51E2 and KLK3, exhibited unexpectedly reduced expression in the majority of advanced states within the TCGA-PRAD dataset (Fig. S10A). Similarly, diverse expression patterns were observed for certain antioncogenes at advanced stages, as exemplified by ENG and TGM2 (Fig. S11A). This discrepancy may arise from the intratumor heterogeneity and histological atypia present in the extracted PCa lesions, which could lead to inconsistencies between the transcriptomic profiles and the pathological diagnoses recorded in the TCGA-PRAD dataset. This comparison underscores that, given the diffusely distributed nature of PCa, ST represents a more appropriate approach for genetic investigation compared to conventional RNA sequencing or related databases.

Furthermore, we evaluated the biological functions of the aforementioned genes. GO and KEGG analyses revealed that oncogenes were significantly enriched in biogenic amine and amine metabolic processes, as well as arginine and proline metabolism, whereas antioncogenes were predominantly associated with extracellular matrix organization and cytoskeleton in muscle cells (Fig. S12A–S12D).

### The proteins encoded by the SLC4A4 and H2AFJ genes exhibit elevated expression levels in advanced-stage PCa tissues

Oncogenes shared by five or more patients were selected as candidates for further validation at the protein level. Among them, several are well-established clinical biomarkers in PCa research, including FOLH1 (PSMA) and AMACR (P504S). Additionally, other shared oncogenes, such as OR51E2 (PSGR), FOS (a subunit of AP-1), and EGR1 (early growth response protein-1), have also been widely reported in PCa studies. Notably, the association of NDUFB9 with malignancy appeared somewhat tenuous, as it was up-regulated in only two out of the three comparative analyses conducted across all five shared tissue samples.

**Figure 7:**
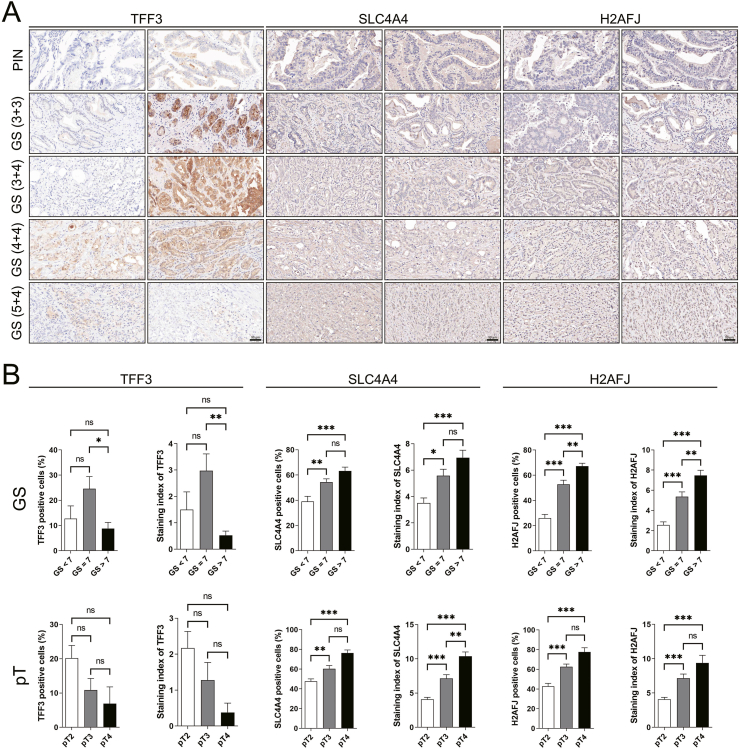
The expression levels of H2AFJ, SLC4A4, and TFF3 proteins in prostate tissues with PIN and varying GSs. **(A)** Representative images of IHC staining for TFF3, SLC4A4, and H2AFJ in prostate cancer (PCa) tissues are presented, illustrating distinct histological classifications, including PIN, GS 3 + 3, GS 3 + 4, GS 4 + 4, and GS 5 + 4. **(B)** The percentages of positively stained cells and the corresponding staining indices for these proteins were quantified and compared across PCa tissues stratified by GSs (GS < 7, GS = 7, and GS > 7) and pT stages (pT2, pT3, and pT4). The results are displayed as histograms, with data represented as the means ± standard errors of the means (SEMs). ns, not significant. *p* > 0.05; ∗*p* < 0.05; ∗∗*p* < 0.01; and ∗∗∗*p* < 0.001.

Based on our screening results and to further investigate novel findings, we subsequently evaluated the protein expression of three candidate genes (TFF3, SLC4A4, and H2AFJ) by IHC in PIN and PCa tissues obtained from a cohort of 90 patients. The clinicopathological characteristics of these patients are summarized in Table 1. IHC staining revealed a gradual increase in the expression of SLC4A4 and H2AFJ proteins correlating with advancing GSs, whereas TFF3 levels remained unchanged (Fig. 7A). The percentages of positively stained cells and staining indices for these proteins were quantified and compared in PCa tissues stratified by GS categories (GS < 7, GS = 7, and GS > 7) and pT stages (pT2, pT3, and pT4). The results indicated a general increase in the expression of SLC4A4 and H2AFJ proteins with the progression of both the GS and pT stages (Fig. 7B), confirming their potential oncogenic roles in PCa. In contrast, TFF3 protein expression was heterogeneous among GE cells and undetectable in a substantial subset of tissues, which precluded robust statistical analysis (Fig. 7A and B). Nonetheless, its reported overexpression in PCa tissues elsewhere suggests a context-dependent role that warrants further investigation.^64^, ^65^, ^66^, ^67^, ^68^, ^69^, ^70^

## Discussion

PCa is a multifocal malignancy originating from GE cells and is characterized by its diffuse distribution throughout prostate tissue,^2^, ^3^, ^4^, ^5^, ^6^, ^7^, ^8^, ^9^, ^10^, ^11^ which complicates visual assessment and pathological diagnosis. ST technology is particularly well-suited for PCa research, as it enables high-resolution, spatially resolved quantification of transcriptomic patterns across histologically distinct structures within the prostate.^16^^,^^29^^,^^31^, ^32^, ^33^, ^34^, ^35^, ^36^ Previous studies have demonstrated that spatial profiles of factor activity identified through ST closely align with those obtained from IHC staining and histological examination.^26^ Building on this foundation, the present study aims to precisely identify genes associated with PCa progression by using ST to analyze DEGs during GS progression in 12 PCa tissue samples.

Analyzing the relationship and origin of multiple lesions within a single sample provides valuable insights into the evolutionary processes underlying PCa. Although current genetic and bioinformatic evidence supports such analyses, it remains insufficient to definitively confirm the multifocal origin of PCa. Some studies have proposed that PCa lesions may originate from independent multiclonal diseases.^17^, ^18^, ^19^, ^20^ This hypothesis is supported by analyses of single nucleotide variants (SNVs)^17^ and allelic imbalances.^18^, ^19^, ^20^ In this study, we also identified transcriptomic heterogeneity across bilateral lesions within the same prostate tissue, further underscoring the complexity of PCa development. However, these prior analyses predominantly focused on a limited subset of prevalent somatic aberrations to infer the dominant clone, which may inadvertently overlook subclonal mutations that are shared among cells due to methodological constraints. Variable patterns of acquired genomic aberrations drive the evolutionary processes of PCa cells, facilitating progression to advanced stages and the emergence of distinct phenotypes within subclones.^12^, ^13^, ^14^, ^15^, ^16^^,^^49^^,^^50^ High-resolution genome-wide analyses in other studies have demonstrated that PCa cells from distinct foci within a single case trace back to a single genomically aberrant precursor, sharing identical allele-specific CNV changes that are stably propagated through cell division.^12^^,^^13^^,^^16^ Therefore, these findings support the theory that disparate lesions in multifocal PCa originate from a monoclonal source derived from a single precursor cell,^12^, ^13^, ^14^, ^15^, ^16^ progressing through an ongoing Darwinian evolutionary process.^71^, ^72^, ^73^

Heterogeneity in Gleason grade has been widely reported in multifocal PCa.^74^ According to the aforementioned monoclonal theory, the heterogeneity of GSs observed across different regions within a single sample may reflect diverse developmental stages of PCa cells originating from a common monoclonal origin. Transcriptomic separation of multiple lesions through PCA and clustering has been shown to be correlated with various pathological subtypes.^26^^,^^29^^,^^31^^,^^32^ In this study, we applied HVGs for dimensionality reduction via PCA to capture major sources of biological variation. Subsequent UMAP and Louvain clustering were then employed to accurately classify tissue spots into distinct histological structures, as demonstrated in our previous methodology.^31^^,^^32^ The resulting classifications were further validated through pathological examination and were annotated based on corresponding histological features.

Although the pathological diagnosis was determined by a senior pathologist, the potential influence of subjective factors necessitates careful consideration to ensure evaluative rigor and reliability. To address this, we computationally validated the accuracy of the annotated histological structures using bioinformatic methods based on both malignancy- and development-related criteria. The mean CNV value (cnv_score) is recognized as a metric for assessing the malignancy of different clusters.^22^^,^^23^ Similarly, DPT (dpt_pseudotime) and PAGA are advanced algorithms for pseudotime and trajectory inference, both of which are implemented within the Scanpy framework.^56^, ^57^, ^58^ In this study, we applied inferCNV, DPT, and PAGA analyses to reconstruct developmental trajectories and progression stages among classified GE regions, including various GSs and PIN. The quantitative results—including cnv_score and dpt_pseudotime, and geodesic distances in the PAGA graph—generally increase with advancing GS, although not uniformly across all samples.

Subsequently, genes associated with PCa progression were identified by performing pairwise comparisons among GE clusters, guided by the preliminary characterization of developmental trajectories and progression stages. The comparison used in this DEG analysis satisfied all criteria for evaluating relative factors across the GS, inferCNV, DPT, and PAGA analyses.

We screened DEGs shared across at least three samples and subsequently evaluated their biological functions through enrichment analysis. Notably, oncogenes were significantly enriched in pathways related to biogenic amine and amine metabolic processes, as well as arginine and proline metabolism. The identified genes included well-established clinical biomarkers of PCa—such as FOLH1, AMACR, and KLK3—all of which showed consistent association with GS progression in at least five PCa tissue samples.

FOLH1 (prostate-specific membrane antigen, PSMA) is a type II transmembrane glycoprotein overexpressed in PCa, with its expression positively correlated with malignancy, metastatic potential, and early recurrence risk.^75^, ^76^, ^77^ It serves as a target for PSMA-PET/CT imaging in detecting primary and metastatic lesions.^78^, ^79^, ^80^ AMACR (P504S) is an IHC marker overexpressed in approximately 90% of PCa cases and high-grade PIN.^81^ Its IHC staining is commonly utilized in conjunction with basal cell markers (such as 34βE12 and P63) for diagnostic confirmation.^82^^,^^83^ KLK3 (prostate-specific antigen, PSA) is the primary serum biomarker for PCa screening and monitoring, aiding in the early detection and assessment of biochemical recurrence (BCR).^84^, ^85^, ^86^, ^87^, ^88^

We identified oncogenes, including TFF3 OR51E2, FOLH1, AMACR, FOS, SLC4A4, EGR1, NDUFB9, and H2AFJ, that were consistently expressed in at least five PCa tissue samples. While not yet established as clinical biomarkers, OR51E2, FOS, and EGR1 have been extensively reported in the context of PCa research and analysis.

OR51E2 (prostate-specific G protein-coupled receptor, PSGR) is exclusively expressed in the human prostate and is overexpressed in PCa, with its expression levels correlated with tumor progression.^89^, ^90^, ^91^, ^92^ It shows potential as a diagnostic biomarker alongside AMACR and PCA3.^93^^,^^94^ Functionally, it promotes inflammation via the interleukin-6 (IL-6) and AKT/NF-κB pathways,^95^, ^96^, ^97^ though β-ionone-induced activation may suppress proliferation via activation of the MAPK family and inhibition of androgen receptor (AR) nuclear translocation.^98^^,^^99^ AP-1 complexes (c-Fos/c-Jun) contribute to PCa progression and therapy resistance.^100^, ^101^, ^102^, ^103^ Fos plays dual roles in PCa: it promotes angiogenesis and neuroendocrine (NE) differentiation,^104^ yet paradoxically suppresses PCa progression and enhances apoptosis in specific contexts.^105^^,^^106^ Although Fos was identified as an oncogene in our ST analysis, its expression was found to be relatively up-regulated in low GS and PIN lesions compared to high GS lesions across Cytassist_1 to Cytassist_5 and Visium_3 samples, underscoring the need for further investigation. EGR1 (early growth response 1) is a key transcription factor that regulates cell growth and survival and promotes PCa progression. It is up-regulated in PCa and associated with high GSs and poor differentiation.^107^^,^^108^ It supports androgen-independent growth and metastasis, and its inhibition reduces tumor incidence in models.^109^, ^110^, ^111^, ^112^

The putative oncogene NDUFB9 exhibited inconsistent and biologically tenuous associations with PCa malignancy, as it was only up-regulated in two of the three comparative analyses in all five tissues. Its functional role in PCa remains unexplored. Based on this limited and contextually distinct evidence, we concluded that NDUFB9 is unlikely to play a major role in PCa development.

To identify novel biomarkers for PCa, we performed IHC analysis to validate the expression of proteins encoded by TFF3, SLC4A4, and H2AFJ in PIN and PCa tissues obtained from 90 patients. The results demonstrated that the protein levels of SLC4A4 and H2AFJ exhibited a progressive increase with GS progression, whereas TFF3 did not exhibit a comparable trend.

TFF3 (trefoil factor 3) is elevated in PCa cells,^64^, ^65^, ^66^, ^67^, ^68^, ^69^, ^70^ an effect potentially attributable to promoter hypomethylation.^69^^,^^70^ Mechanistically, TFF3 contributes to prostate carcinogenesis by blocking mitochondria-mediated apoptosis.^64^ However, the association between TFF3 expression and GS progression remains controversial. While one study reported elevated TFF3 protein expression in tumors with a GS > 7 ^67^, others found no correlation or even a negative association with the GS, tumor stage, or recurrence rates.^65^^,^^66^^,^^70^^,^^113^^,^^114^ Notably, our IHC validation revealed that TFF3 protein was undetectable in a substantial subset of PCa tissues, including high-grade (GS 5 + 4) cases, complicating robust statistical analysis. Although TFF3 expression has been used for patient stratification to evaluate its progression and prognostic role in PCa, definitive clinical conclusions remain elusive.^65^, ^66^, ^67^^,^^113^^,^^114^

H2AFJ (H2A histone family member J) is significantly overexpressed in luminal epithelial gland cells, including in breast cancer and PCa tissues.^115^ Similarly, SLC4A4 (solute carrier family 4 member 4) is up-regulated in PCa and promotes disease progression; its inhibition suppresses PCa cell proliferation, migration, invasion, and enzalutamide resistance in preclinical models.^116^, ^117^, ^118^, ^119^ Integrated with our findings, these results position SLC4A4 and H2AFJ as promising candidate biomarkers for prostate cancer, warranting further functional and clinical validation.

This study also revealed that well-known oncogenes, including OR51E2, KLK3, and KLK2, which were identified via ST analysis, exhibit negative associations with advanced disease stages in the TCGA-PRAD dataset. This discrepancy underscores inconsistencies between transcriptomic profiles and pathological diagnoses within the TCGA-PRAD, highlighting the potential of ST as a superior predictive tool for genetic research in PCa compared to conventional RNA sequencing and related databases.

## Conclusions

In summary, we leveraged ST to comprehensively analyze the transcriptome profiles of 12 PCa samples exhibiting diverse GE histological structures. Through the integration of pairwise comparisons of GE clusters with developmental trajectories and progression stages of classified tumor regions, we identified key genes driving PCa progression. IHC validation further confirmed the elevated expression of SLC4A4 and H2AFJ in advanced GS. Overall, this study establishes an ST-based framework for predicting PCa progression and nominates promising biomarker candidates for clinical translation.

## CRediT authorship contribution statement

**Yongjun Quan:** Writing – original draft, Validation, Supervision, Project administration, Methodology, Investigation, Formal analysis, Data curation. **Mingdong Wang:** Validation, Supervision, Formal analysis, Data curation. **Fan Zou:** Data curation. **Hong Zhang:** Methodology. **Yishan Zhang:** Validation. **Yongchen Jin:** Supervision. **Hao Ping:** Writing – original draft, Visualization, Validation, Supervision, Funding acquisition, Formal analysis.

## Ethics declaration

Ethical approval was granted by the Ethics Committee of Beijing Tongren Hospital, affiliated with Capital Medical University (Approval No.: TREC2023-KY030). Written informed consent was obtained from all participants included in this study.

## Data availability

The data and materials supporting the conclusions of this study are included within the article and its supplementary materials. All spatial transcriptomic sequencing data generated in this work have been deposited in the Science Data Bank (ScienceDB). Specifically, the Visium CytAssist ST data can be accessed at https://cstr.cn/31253.11.sciencedb.25452 with the corresponding DOI: https://doi.org/10.57760/sciencedb.25452. The standard Visium ST data are available at https://cstr.cn/31253.11.sciencedb.09389 and https://doi.org/10.57760/sciencedb.09389.

## Funding

This work was supported by the National Natural Science Foundation of China (No. 82272864 to Hao Ping) and the Capital's Funds for Health Improvement and Research (No. 2024-2-2059 to Hao Ping).

## Conflict of interests

The authors declare no competing interests.
