# Supplementary material for: Uncovering genes driving developmental stage progression in prostate cancer through spatial transcriptomics
Source: Genes Dis. 2025 Dec 11;13(5):101983. doi: 10.1016/j.gendis.2025.101983 (PMC13254596; doi:10.1016/j.gendis.2025.101983)
Supplement: Multimedia component 1 [file mmc1.pdf]

Supplementary figures

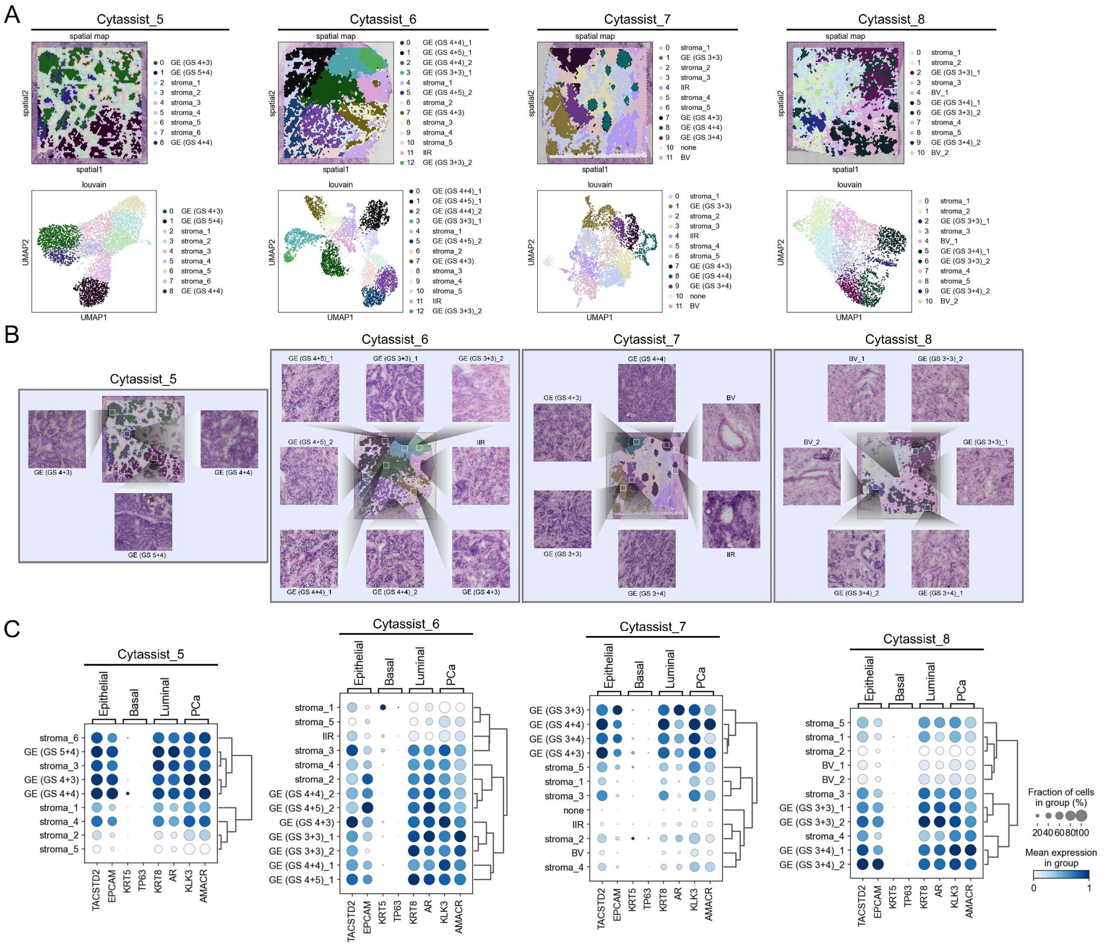

Figure S1. The transcriptomic classification of all spatially resolved spots across CytAssist_5 to CytAssist_8 PCa tissue sections into distinct histological structures.

(A) Transcriptomic classifications across all spots were performed using PCA, UMAP, and Louvain clustering analysis. The resulting clusters were overlaid onto the histological image and annotated according to their corresponding histological structures (upper panel), and visualized in a UMAP embedding (lower panel). (B) The regions of distinct clusters were magnified and pathologically evaluated based on corresponding H&E-stained images from each PCa section. (C) The expression of canonical marker genes—including TACSTD2 and EPCAM (epithelial), KRT5 and TP63 (basal), KRT8 and AR (luminal), and KLK3 and AMACR (PCa) —was analyzed across annotated clusters and visualized as bubble plots.

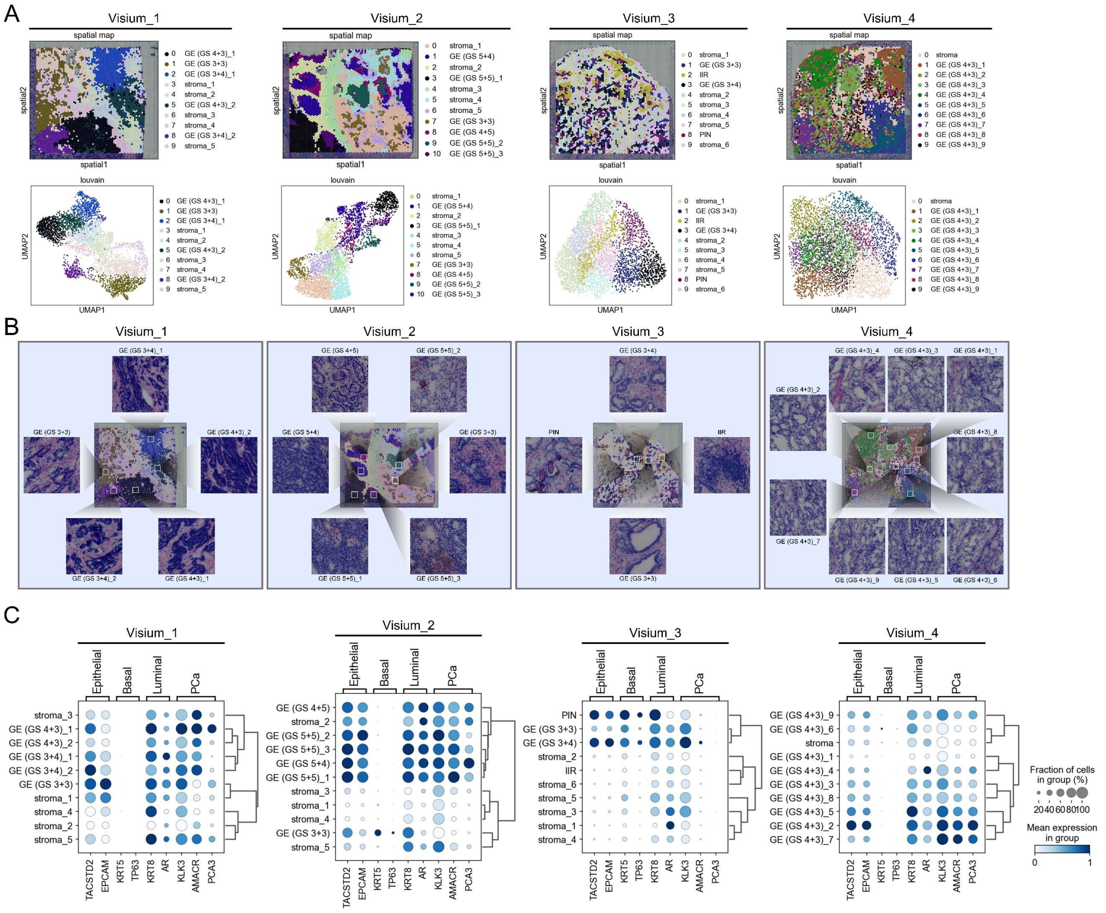

Figure S2. The transcriptomic classification of all spatially resolved spots across Visium_1 to Visium_4 PCa tissue sections into distinct histological structures.

(A) Transcriptomic classifications across all spots were performed using PCA, UMAP, and Louvain clustering analysis. The resulting clusters were overlaid onto the histological image and annotated according to their corresponding histological structures (upper panel), and visualized in a UMAP embedding (lower panel). (B) The regions of distinct clusters were magnified and pathologically evaluated based on corresponding H&E-stained images from each PCa section. (C) The expression of canonical marker genes—including TACSTD2 and EPCAM (epithelial), KRT5 and TP63 (basal), KRT8 and AR (luminal), and KLK3, AMACR, and PCA3 (PCa) —was analyzed across annotated clusters and visualized as bubble plots.

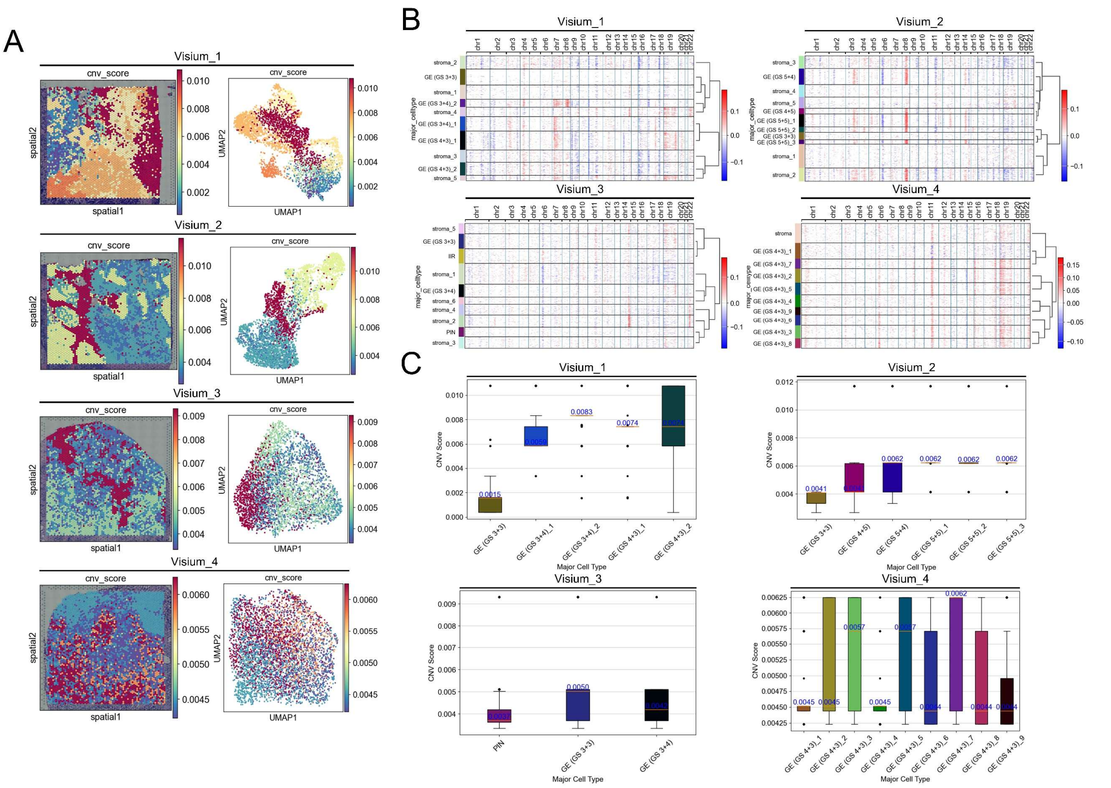

Figure S3. Evaluation of tumor malignancy in varying GE regions across Visium_1 to Visium_4 PCa tissue sections using inferCNV analysis.

(A) The CNV scores were calculated for spatially resolved spots across four samples using inferCNV. The scores are visualized as spatial activity maps overlaid on the histological images (left panel) and as UMAP embeddings (right panel). (B) The chromosomal landscapes derived from inferCNV values for distinguishing histological clusters across the samples are displayed separately as corresponding heatmaps. (C) The median CNV scores, along with their IQRs, for GE clusters are summarized using boxplots for each sample.

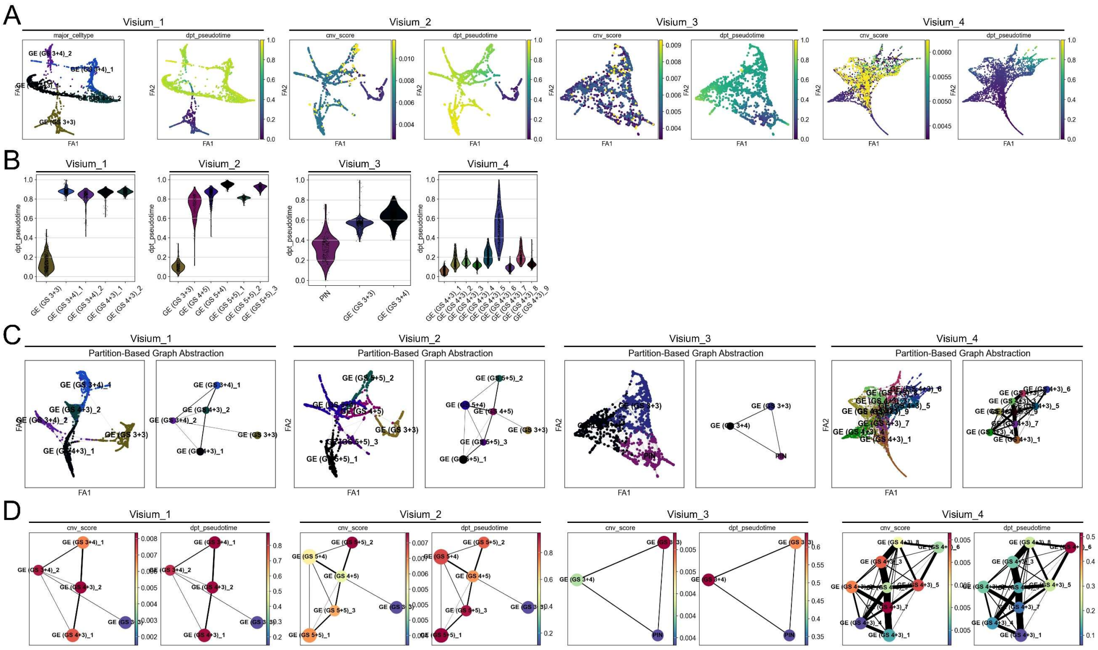

Figure S4. Prediction of progression stages and developmental trajectories across Visium_1 to Visium_4 PCa tissue sections.

(A) Developmental progression was reconstructed using DPT analysis. Diffusion maps of spatially resolved spots for each sample are presented, displaying coordinates specific to varying GE clusters (left panel) and corresponding dpt_pseudotime values (right panel). (B) The quantitative distribution of dpt_pseudotime values within GE clusters for each sample is visualized as violin plots. (C) Developmental trajectories of GE clusters for each sample are visualized using PAGA graphs, displaying individual spatial spots (left panel) and GE clusters (right panel). (D) The CNV score (left panel) and dpt_pseudotime values (right panel) were integrated into the PAGA graph to illustrate developmental dynamics within each GE cluster.

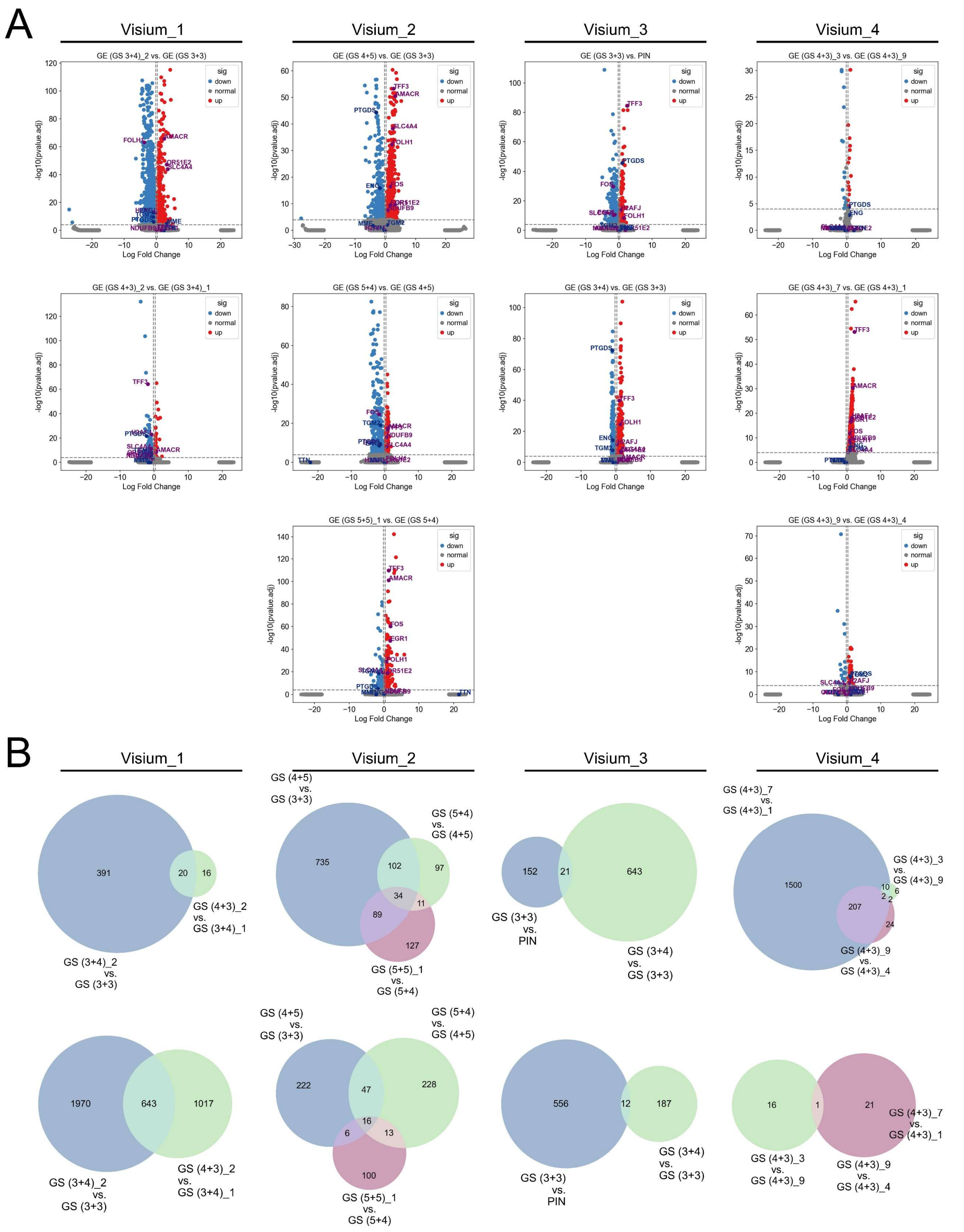

Figure S5. DEG analysis across varying clusters based on progression stages and developmental trajectories in Visium_1 to Visium_4 PCa tissue sections.

(A) DEG analysis was performed for GE clusters, ensuring that the comparison objects exhibited consistent relative progression stages and developmental trajectories. The comparisons for each tissue section are illustrated using volcano plots, with key genes highlighted: oncogenes (TFF3, OR51E2, FOLH1, AMACR, FOS, SLC4A4, EGR1,

NDUFB9, and H2AFJ) in dark magenta and antioncogenes (MME, PTGDS, TTN, ENG, and TGM2) in dark blue. (B) The numbers of genes that were significantly and concurrently upregulated (upper panel) or downregulated (lower panel) in the above comparisons for each tissue section are depicted in Venn diagrams.

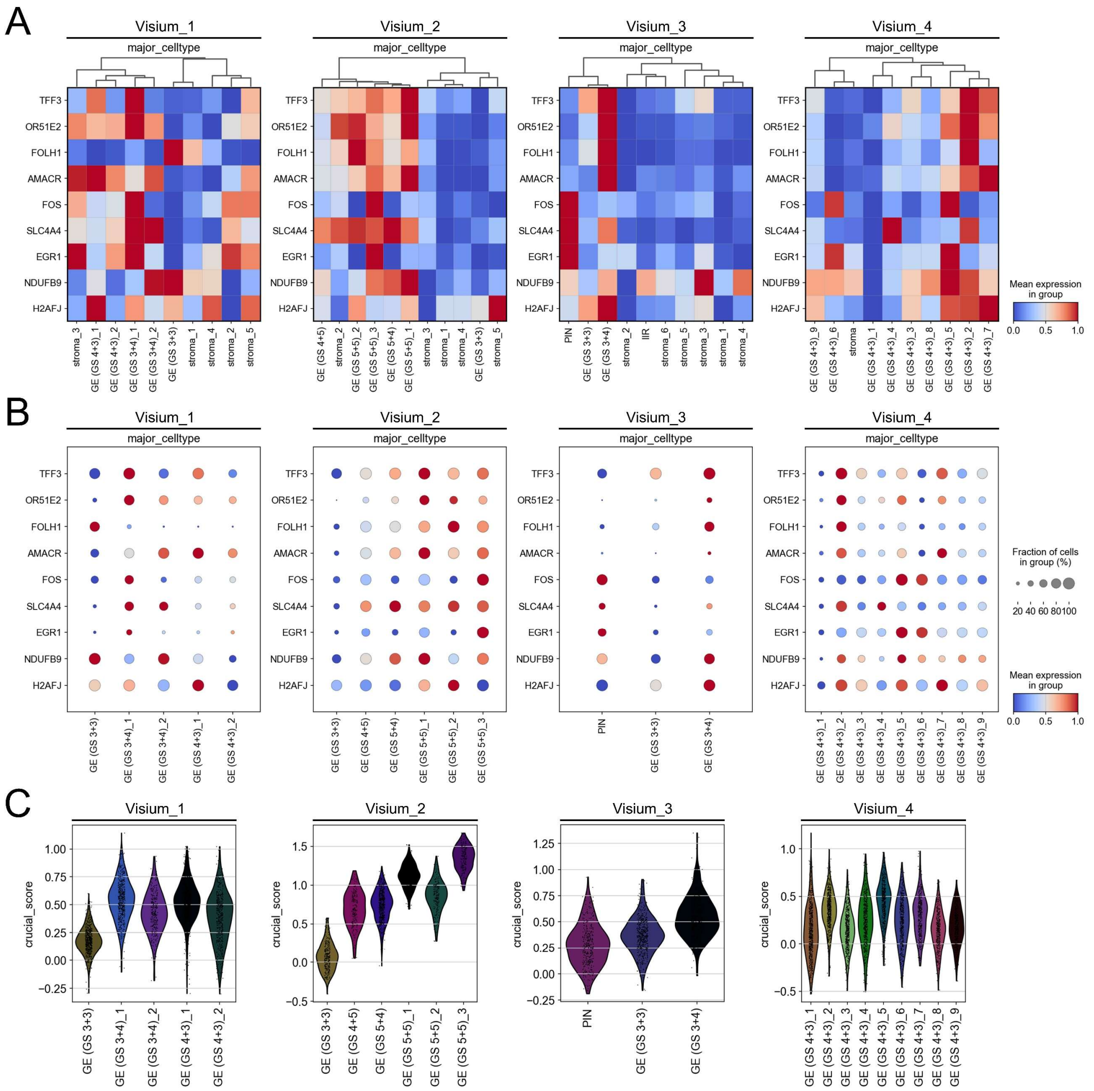

Figure S6. The expression patterns of key oncogenes across the varying histological structures in the Visium_1 to Visium_4 PCa tissue sections.

(A) The expression patterns of oncogenes shared by at least five tissue sections within distinct histological structures of each tissue are visualized as heatmaps. (B) The expression patterns of these genes within distinct GE structures of each tissue are displayed as bubble plots. (C) Gene scores (crucial_scores) derived from the identified 9 oncogenes were calculated, and their distributions across varying GE clusters in each tissue section are visualized as violin plots.

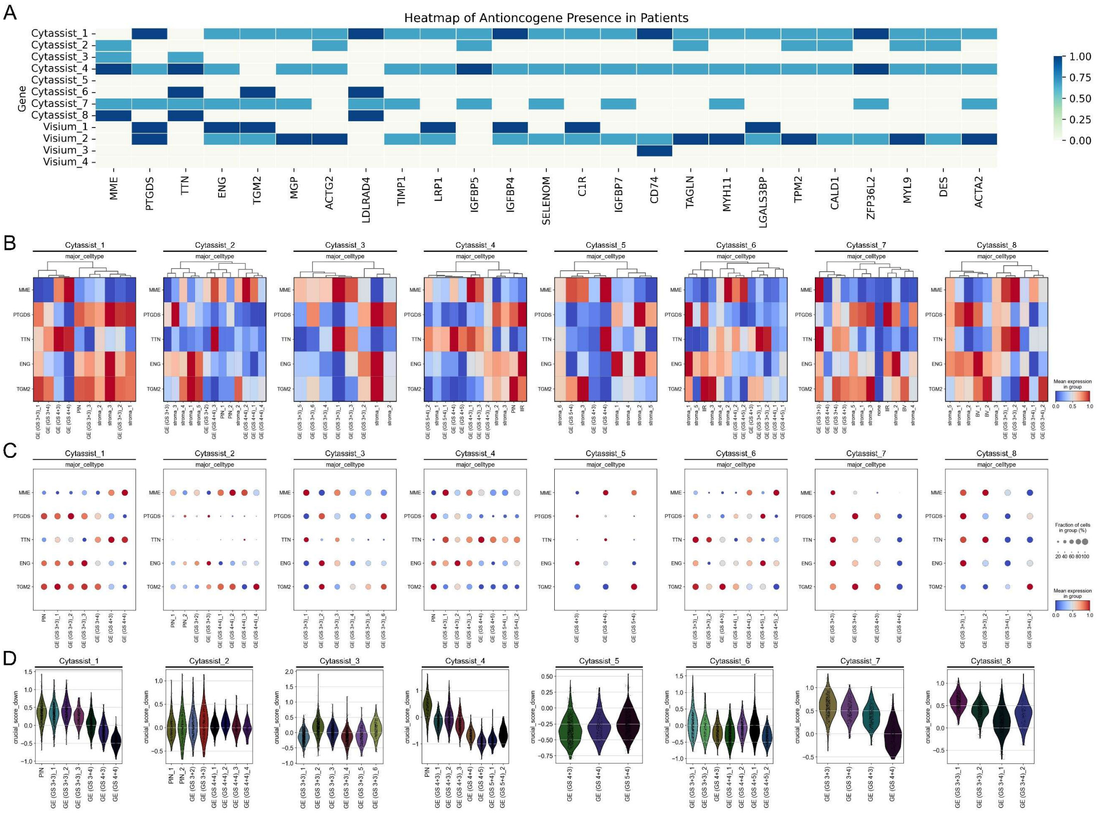

Figure S7. Identifying key antioncogenes associated with the progression of developmental stages in 12 PCa tissue sections.

(A) Significant DEGs intersected at least two comparisons within each tissue are summarized and visualized as heatmap. Each gene is assigned a value of 1 (dark blue) if consistently upregulated across all comparisons within a sample, 0.66 (light blue) if upregulated in two of three comparisons, and 0 (light grey) otherwise. (B) The expression patterns of antioncogenes shared by at least five tissue sections within clusters corresponding to the distinct histological structures of each tissue are visualized as heatmaps. (C) The expression patterns of these genes within distinct GE structures of each tissue are displayed as bubble plots. (D) Gene scores (crucial_scores) derived from the identified 5 antioncogenes were calculated, and their distributions across varying GE clusters in each tissue section are visualized as violin plots.

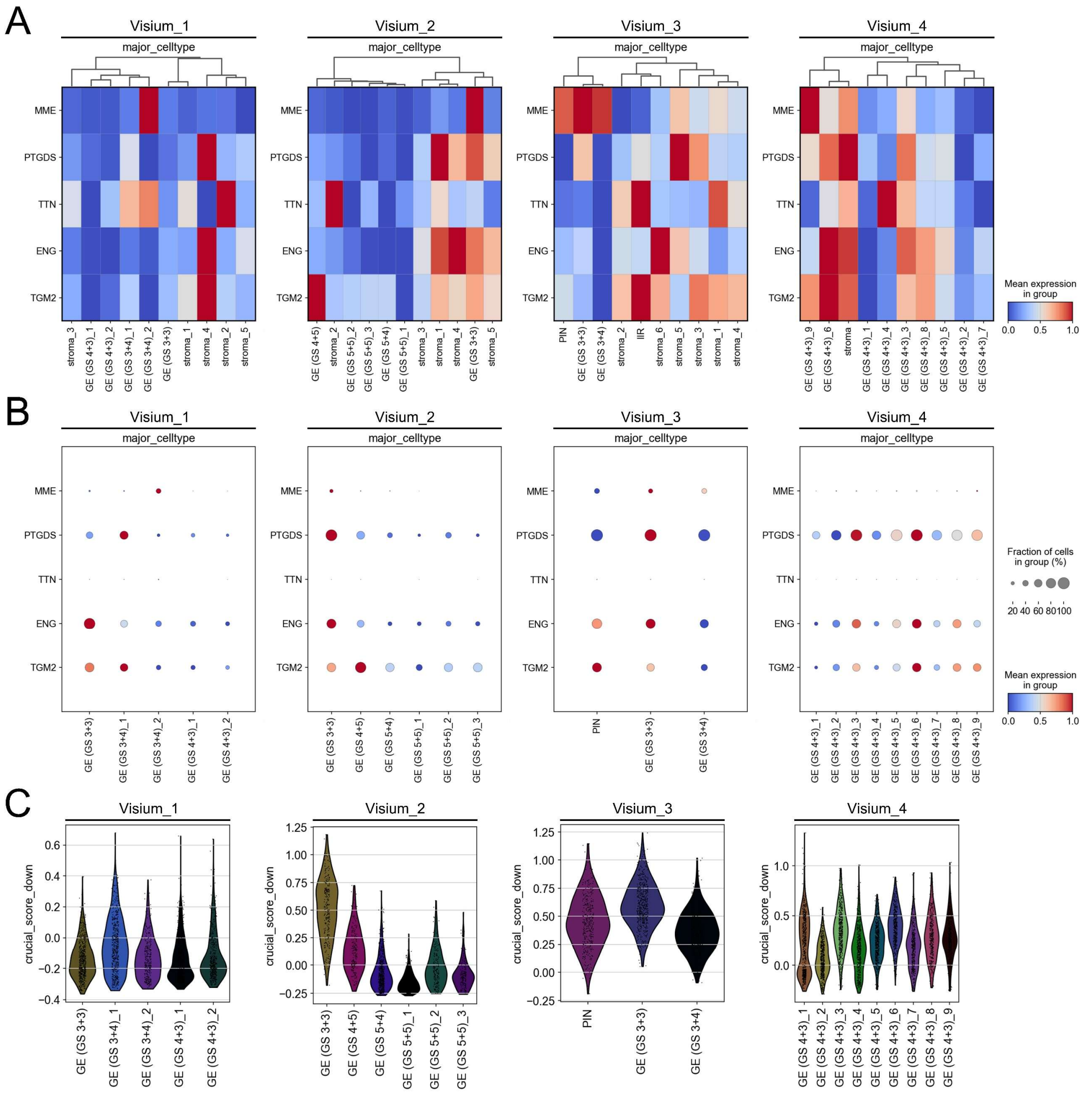

Figure S8. The expression patterns of key antioncogenes across the varying histological structures in the Visium_1 to Visium_4 PCa tissue sections.

(A) The expression patterns of antioncogenes shared by at least five tissue sections within distinct histological structures of each tissue are visualized as heatmaps. (B) The expression patterns of these genes within distinct GE structures of each tissue are displayed as bubble plots. (C) Gene scores (crucial_scores) derived from the identified 5 antioncogenes were calculated, and their distributions across varying GE clusters in each tissue section are visualized as violin plots.

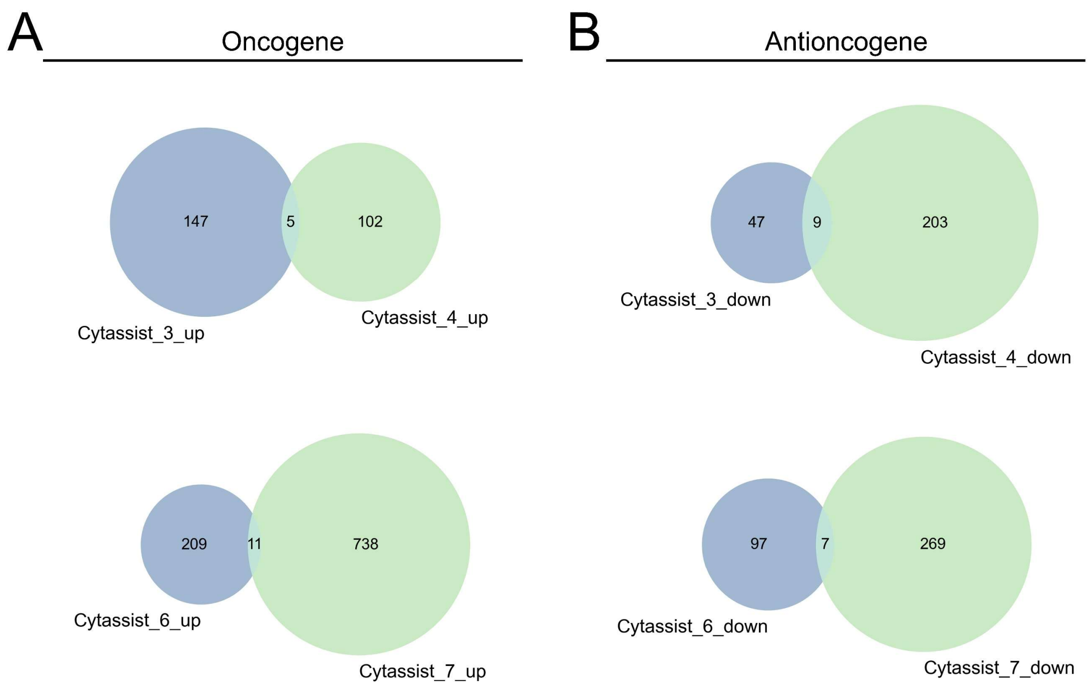

Figure S9. The number of shared genes among different samples extracted from the same prostate.

(A and B) The samples Cytassist_3 and Cytassist_4, as well as Cytassist_6 and Cytassist_7, were respectively obtained from bilateral lesions of prostate tissue. The shared oncogenes (A) and antioncogenes (B) for each pair are visualized in Venn diagrams.

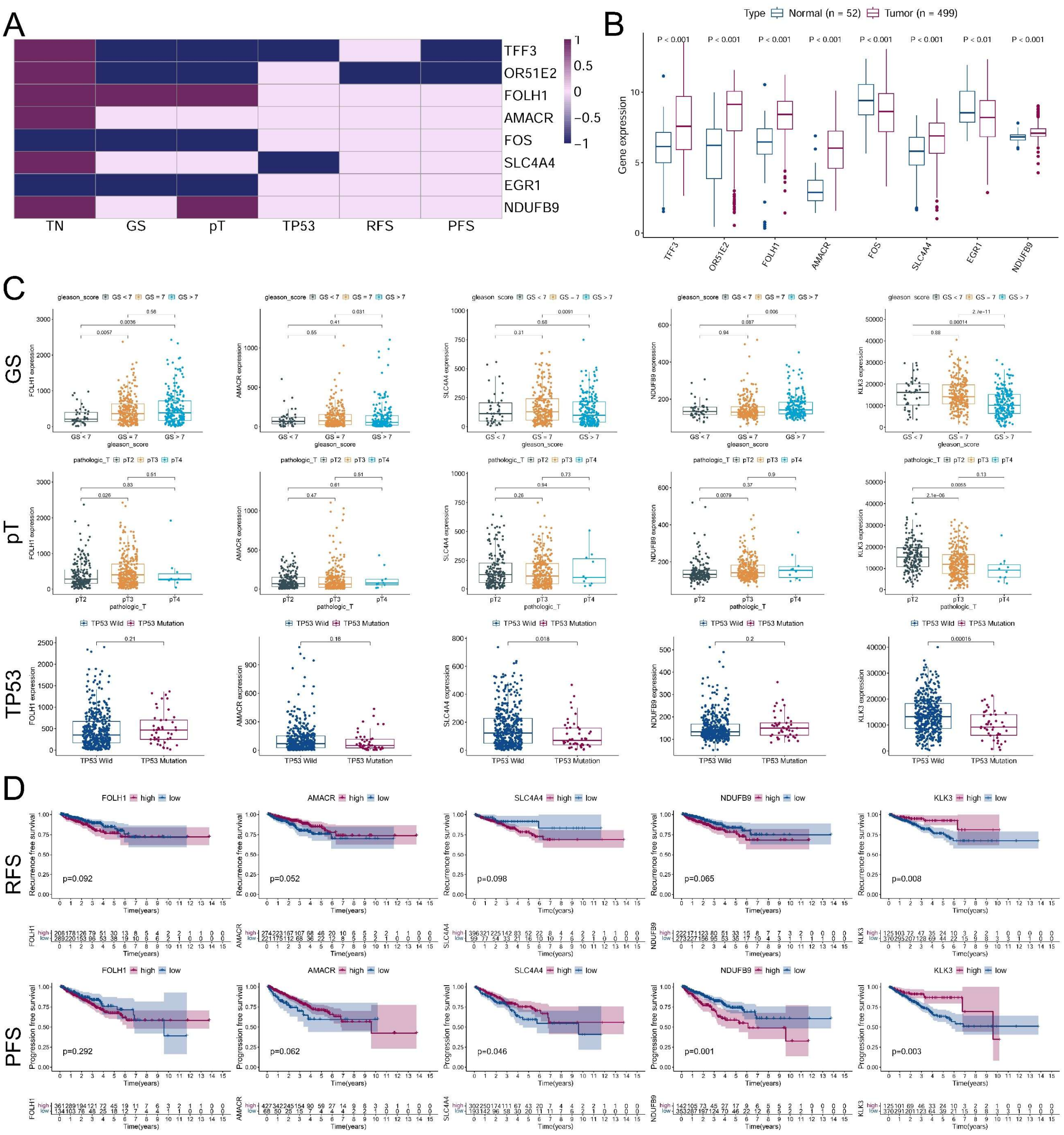

Figure S10. Validation of identified key oncogenes using the TCGA-PRAD dataset.

(A) The expression patterns of 8 oncogenes (excluding H2AFJ, which could not be identified due to genomic alignment limitation with the TCGA-PRAD dataset) are presented as a heatmap across various clinicopathological and prognostic comparisons. Each gene is assigned a value based on its expression status: 1 (deep purplish-red) for upregulation, 0 (light pinkish-purple) for no significant change, and -1 (dark blue) for downregulation in the respective comparisons. The horizontal axis represents the comparisons, including tumor versus normal tissue (TN), GS > 7 versus GS < 7 (GS), pT3 versus pT2 (pT), TP53-mutated versus wild-type (TP53), and prognostic significance of the gene expression status (high versus low) for RFS and PFS (RFS and PFS). The vertical axis lists the oncogenes. (B) The expression levels of these oncogenes in PCa and normal prostate tissues from the TCGA-PRAD dataset are

displayed in a boxplot, with the median and IQR values indicated. (C) The distribution patterns of five representative genes (FOLH1, AMACR, SLC4A4, NDUFB9, and KLK3) are analyzed across tumor regions with different GSs (GS < 7, GS = 7, and GS > 7), pT stages (pT2, pT3, and pT4), and TP53 mutation status (mutation and wild-type) in the TCGA-PRAD dataset. The data are presented as boxplots showing median and IQR values, with Wilcoxon test P-values presented above each pairwise comparison. (D) Kaplan–Meier survival analyses were performed to evaluate the prognostic significance of the five genes for RFS and PFS. TCGA-PRAD patients were stratified into two groups based on the optimal cutoff value for each gene. The results are visualized using Kaplan−Meier survival curves, which present P-values and the corresponding numbers at risk.

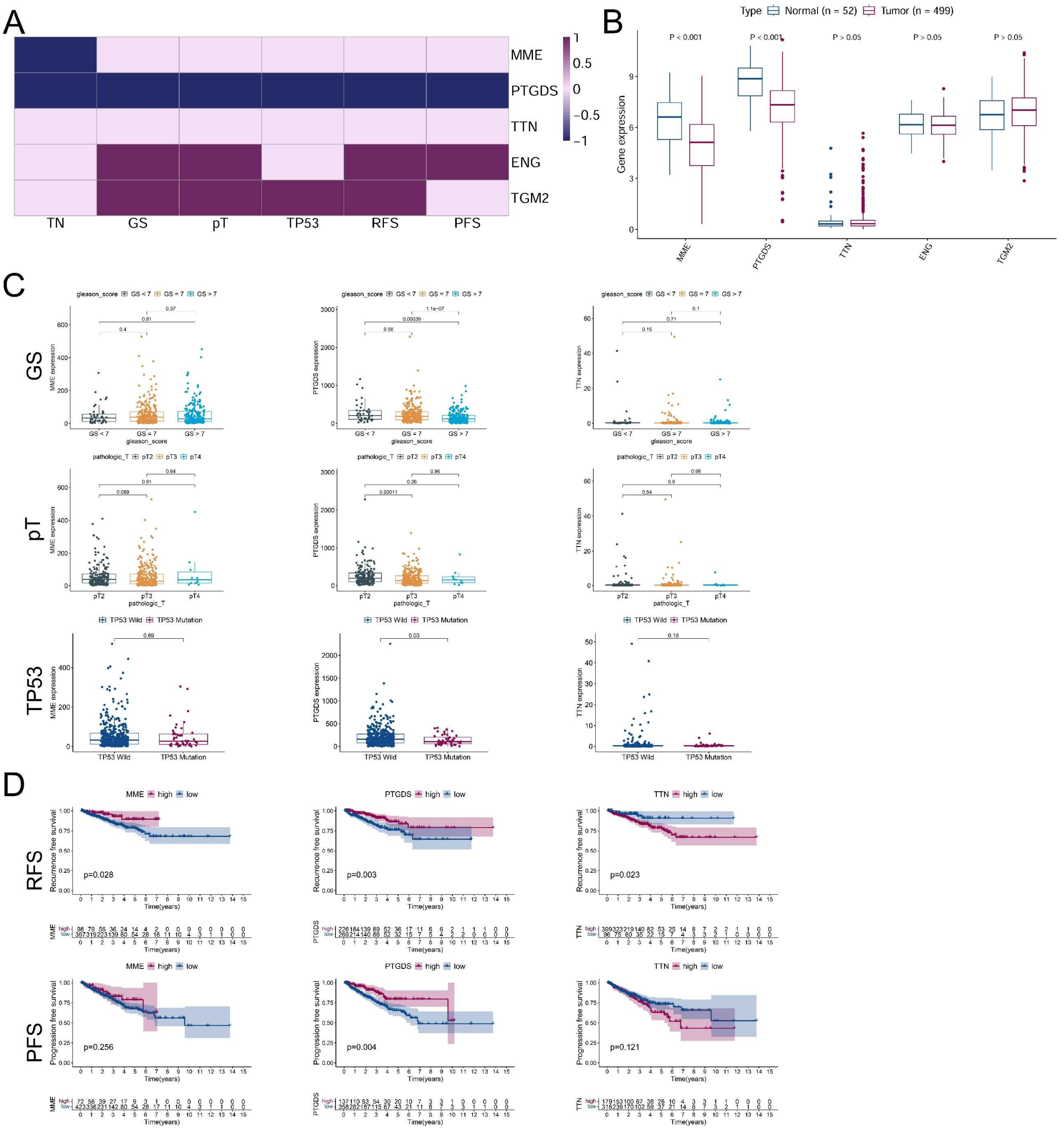

Figure S11. Validation of identified key antioncogenes using the TCGA-PRAD dataset.

(A) The expression patterns of 5 antioncogenes are presented as a heatmap across various clinicopathological and prognostic comparisons. Each gene is assigned a value based on its expression status: 1 (deep purplish-red) for upregulation, 0 (light pinkish-purple) for no significant change, and -1 (dark blue) for downregulation in the respective comparisons. The horizontal axis represents the comparisons, including tumor versus normal tissue (TN), GS > 7 versus GS < 7 (GS), pT3 versus pT2 (pT), TP53-mutated versus wild-type (TP53), and prognostic significance of the gene expression status (high versus low) for RFS and PFS (RFS and PFS). The vertical axis lists the oncogenes. (B) The expression levels of these antioncogenes in PCa and normal prostate tissues from the TCGA-PRAD dataset are displayed in a boxplot, with the median and IQR values indicated. (C) The distribution patterns of three representative genes (MME, PTGDS, and TTN) are analyzed across tumor regions with different GSs (GS < 7, GS = 7, and GS > 7), pT stages (pT2, pT3, and pT4), and TP53 mutation status (mutation and wild-type) in the TCGA-PRAD dataset. The data are presented as boxplots showing median and IQR values, with Wilcoxon test P-values presented above each pairwise comparison. (D) Kaplan–Meier survival analyses were performed to evaluate the prognostic significance of the four genes for RFS and PFS. TCGA-PRAD patients were stratified into two groups based on the optimal cutoff value for each gene. The results are visualized using Kaplan−Meier survival curves, which present P-values and the corresponding numbers at risk.

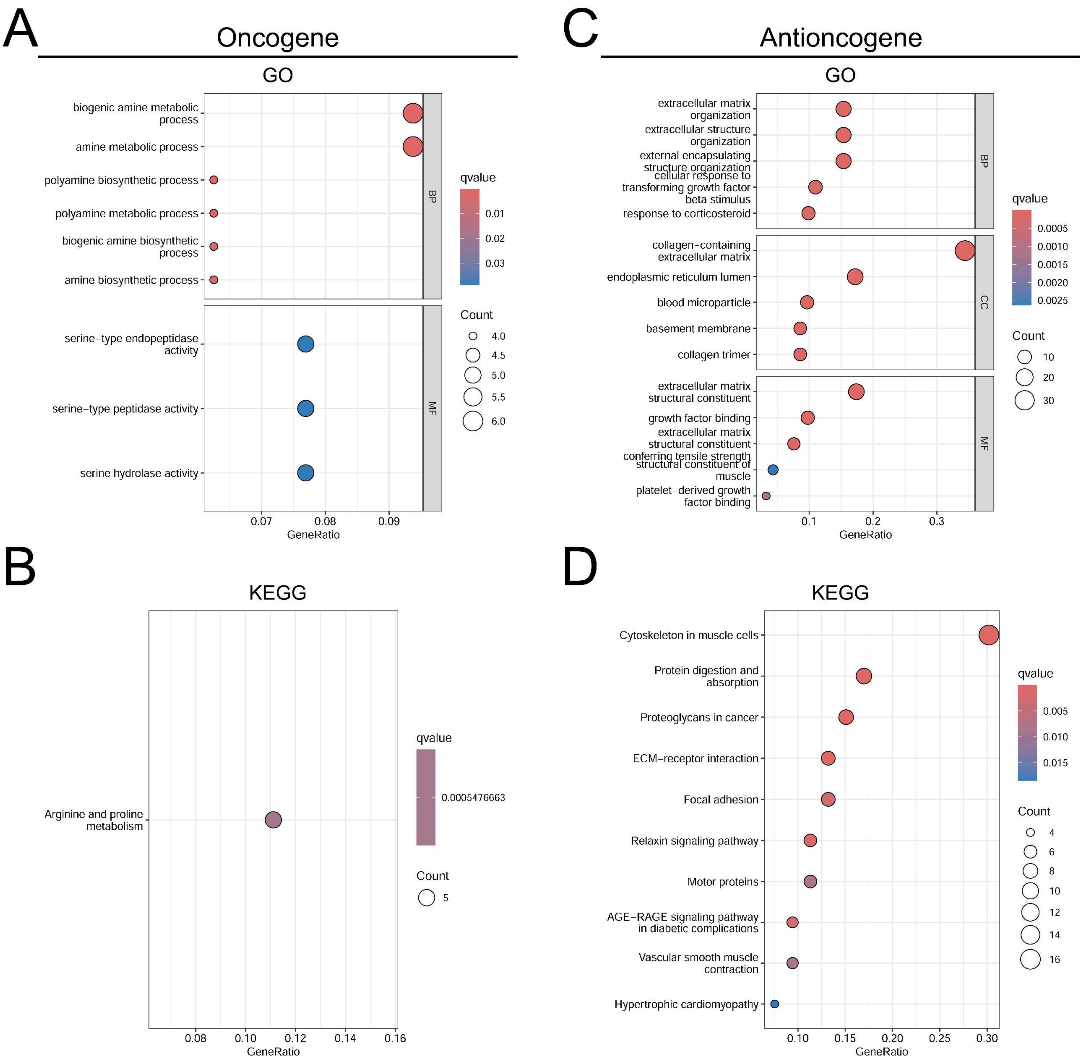

Figure S12. Evaluation of the biological functions of the key genes through enrichment analyses.

(A to D) The oncogenes (A and B) and antioncogenes (C and D) shared by at least three samples were analyzed through GO (up) and KEGG (down) enrichment analyses. The results are visualized as dot plots. GO categories encompass BP (biological process), CC (cellular component), and MF (molecular function).

Supplementary table

Supplementary Table 1. Information of Relative antibodies.

Protein	Brand	NO.
H2AFJ	ThermoFisher	PA5-117468
SLC4A4	Abcam	ab187511
TFF3	Abcam	ab109104
β-actin	CST	#3700

CST: Cell Signaling Technology.
